# Application of Surface-Modified Nanoclay in a Hybrid Adsorption-Ultrafiltration Process for Enhanced Nitrite Ions Removal: Chemometric Approach vs. Machine Learning

**DOI:** 10.3390/nano13040697

**Published:** 2023-02-10

**Authors:** Corneliu Cojocaru, Petronela Pascariu, Andra-Cristina Enache, Alexandra Bargan, Petrisor Samoila

**Affiliations:** 1 Laboratory of Inorganic Polymers, “Petru Poni” Institute of Macromolecular Chemistry, 41A Grigore Ghica Voda Alley, 700487 Iasi, Romania; 2 Laboratory of Physical Chemistry of Polymers, “Petru Poni” Institute of Macromolecular Chemistry, 41A Grigore Ghica Voda Alley, 700487 Iasi, Romania

**Keywords:** nanoclay, adsorption, ultrafiltration, nitrite removal, modeling, machine learning

## Abstract

Herein, we report the results of a study on combining adsorption and ultrafiltration in a single-stage process to remove nitrite ions from contaminated water. As adsorbent, a surface-modified nanoclay was employed (i.e., Nanomer^®^ I.28E, containing 25–30 wt. % trimethyl stearyl ammonium). Ultrafiltration experiments were conducted using porous polymeric membranes (Ultracel^®^ 10 kDa). The hybrid process of adsorption-ultrafiltration was modeled and optimized using three computational tools: (1) response surface methodology (RSM), (2) artificial neural network (ANN), and (3) support vector machine (SVM). The optimal conditions provided by machine learning (SVM) were found to be the best, revealing a rejection efficiency of 86.3% and an initial flux of permeate of 185 LMH for a moderate dose of the nanoclay (0.674% *w*/*v*). Likewise, a new and more retentive membrane (based on PVDF-HFP copolymer and halloysite (HS) inorganic nanotubes) was produced by the phase-inversion method, characterized by SEM, EDX, AFM, and FTIR techniques, and then tested under optimal conditions. This new composite membrane (PVDF-HFP/HS) with a thickness of 112 μm and a porosity of 75.32% unveiled an enhanced rejection efficiency (95.0%) and a lower initial flux of permeate (28 LMH). Moreover, molecular docking simulations disclosed the intermolecular interactions between nitrite ions and the functional moiety of the organonanoclay.

## 1. Introduction

Anionic species such as nitrite (NO_2_^−^) and nitrate (NO_3_^−^) play an essential role in the biogeochemical cycle of nitrogen in nature. However, high nitrite and nitrate concentrations in natural waters are very toxic to human health [1]. Generally, nitrite ions are more hazardous than nitrate ions. Therefore, the maximum admissible concentration for nitrite (NO_2_^−^) is about 100-fold less than that of nitrate (NO_3_^−^). For instance, according to the European Council Directive (98/83/EC), the maximum allowed concentration for nitrate (NO_3_^−^) in drinking water was established at 50 mg/L, whereas for nitrite (NO_2_^−^) the maximum admissible concentration was recommended at a much lower level of 0.5 mg/L [2]. The high toxicity of nitrite is related to the ability of NO_2_^−^ anions to readily interact with amines and to form carcinogenic compounds known as nitrosamines [3,4,5].

Nitrite and nitrate salts are used in many domains such as the food industry, agriculture, and the chemical industry [6]. Consequently, the pollution of natural waters (rivers and groundwater) with nitrogen-based oxoanions frequently occurs as a result of these agricultural and industrial activities [5,6,7]. So far, different methods have been employed to remove nitrites and nitrates from contaminated waters, including selective ion exchange [8], adsorption [9], biosorption [10], reverse osmosis [11], electrodialysis [12], and biological denitrification [13]. Among these methods, adsorption is the most attractive one owing to its simplicity, lower operating costs, and relevant efficiency. In this respect, different adsorbent materials were reported in the applications dealing with removing of nitrite and nitrate ions from contaminated waters. To be specific, the sepiolite (clay mineral) and modified sepiolite were tested for nitrite and nitrate removal from aqueous solutions [14,15,16]. The adsorption capacity of pristine sepiolite was reported as being 0.65 mg/g for retention of nitrite [14] and 3.4 mg/g for retention of nitrate [15]. Previous studies [16,17] reported that the chemical modification of clays with surfactants was favorable. The obtained organoclays showed a higher adsorption capacity for the retention of nitrate [16,17].

It is well known that in the adsorption process, the final step implies the separation of the solid and liquid phases. When a powder adsorbent is used, the separation step is often realized by intense centrifugation, which consumes energy at a high level. Therefore, a robust alternative is to integrate the adsorption with the filtration through the membrane (e.g., low-pressure driven microfiltration or ultrafiltration) in a one-step process. Combining adsorption and ultrafiltration (UF) in a hybrid process demonstrated some benefits in water purification [18] and wastewater treatment [19]. For example, the association of the adsorption and UF in a one-step process resulted in the better physical removal of the dissolved pollutants from aqueous solutions [18]. Hence, the application of the UF-adsorption hybrid process to remove nitrogen-based oxoanions from contaminated waters is of practical interest.

Currently, it has become essential to explore and optimize the investigated processes using modeling and computer-aided simulation techniques. Modern modeling and computational tools are aimed to advance the level of research within the field. In modeling a real system or process, one tries to find the true response surface function, which is a complex relation of dependency between input variables (factors) and the output variable (response). However, the true response function can only be approximated or estimated by different data-driven modeling approaches such as response surface methodology (RSM), artificial neural networking (ANN), support vector machine (SVM), and others. Recent advancements in data-driven modeling of separation processes in water purification and wastewater treatment have highlighted the importance of modern modeling tools like RSM, ANN, and SVM [20]. The last two (i.e., ANN and SVM) are part of machine learning (ML), which is a type of artificial intelligence (AI) that uses historical data as input to predict new output values for a studied system or process.

Three main objectives were taken into account in this paper. The first one aimed to study the adsorption-ultrafiltration hybrid process applied to remove nitrite (NO_2_^−^) anions from aqueous solutions using organoclay as an adsorbent and a porous polymeric membrane as a separation barrier. The second objective dealt with data-driven modeling and model-based optimization of the adopted adsorption-ultrafiltration hybrid process. In this respect, the response surface methodology (RSM), artificial neural network (ANN), and a support vector machine (SVM) were employed as modeling tools to point out the common and distinct features among these modeling methods. The third objective was to assess a new composite polymeric membrane (prepared by the phase-inversion method) under the established optimal conditions.

## 2. Materials and Methods

### 2.1. Materials

Sodium nitrite (NaNO_2_), acquired from Merck Millipore (Darmstadt, Germany), was used for the preparation of aqueous solutions loaded with nitrite (NO_2_^−^) ions. Griess’ reagent (Sigma-Aldrich, St. Louis, MO, USA) was employed for detecting nitrite ions in aqueous solutions via diazotization reaction. As an adsorbent for capturing the nitrite ions, surface-modified nanoclay (organoclay) was applied. This nanoclay is a commercial product (Nanomer^®^ clay I.28E) that was purchased from Sigma-Aldrich. Note that this product (Nanomer^®^ I.28E), containing 25–30 wt. % trimethyl stearyl ammonium TmSA (C_21_H_46_N^+^), relies on the montmorillonite (MMT) clay matrix that has been surface modified. Sulphuric acid (H_2_SO_4_) 98% (Carl Roth, Karlsruhe, Germany) and sodium hydroxide (NaOH) ≥98% (Sigma-Aldrich) were employed for the preparation of 0.1 M H_2_SO_4_ and 0.1 M NaOH solutions, respectively, which were used for pH adjustments.

Commercial polymeric porous membranes (Ultracel^®^ from Merck Millipore) with 10 kDa molecular weight cut-off (MWCO) were employed to perform the basic ultrafiltration assays according to the design of the experiments. In addition, a composite porous membrane (flat-sheet) was produced and tested in laboratory conditions. To this end, the following products were acquired: Poly(vinylidene fluoride-co-hexafluoropropylene) (PVDF-HFP) with an average molecular mass of M_n_ ≈ 130 kDa (M_w_/M_n_ ≈ 3.1) was supplied by Sigma-Aldrich; Halloysite (H_4_Al_2_O_9_Si_2_∙2H_2_O) nanotubes with dimensions of approximately 30 to 70 nm × 1.4 μm (Sigma-Aldrich) were used as inorganic nanofillers for the composite membrane; As a pore generator (porogen), the polyethylene glycol PEG-400 (Aldrich) was adopted; As the aprotic solvent, N,N-Dimethylacetamide (DMAC) from Sigma-Aldrich was employed for composite membrane fabrication through the phase-inversion method. Isopropyl alcohol (Chemical Company, Iasi, Romania) was used in assays for porosity estimation.

### 2.2. Experimental Methods

**Figure 1:**
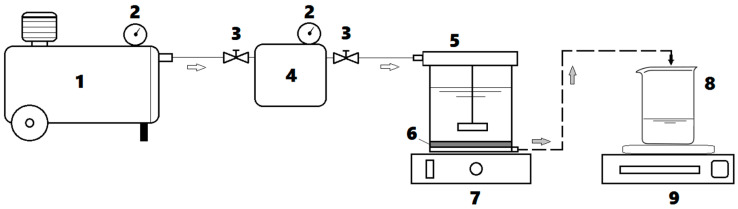
Dead-end ultrafiltration (UF) system employed for experimentation: (**1**) air compressor; (**2**) manometers; (**3**) pressure control valves; (**4**) dispensing pressure vessel; (**5**) UF stirred cell; (**6**) porous membrane; (**7**) magnetic stirrer; (**8**) permeate container; (**9**) digital balance.

Ultrafiltration (UF) assays were carried out in a dead-end flow regime by using an Amicon^®^ Stirring-Cell (Merck Millipore) of 50 mL volumetric capacity. In this regard, the commercial flat-sheet membranes (Ultracel^®^ 10 kDa, Merck Millipore) were applied, possessing the following characteristics: (1) material: regenerated cellulose; (2) molecular weight cut-off (MWCO) of 10 kDa; (3) membrane diameter of 4.45 cm; and (4) effective filtration area equal to 13.4 cm^2^. The experimental setup for the ultrafiltration system is depicted in Figure 1.

In the course of the adsorption-ultrafiltration experiments, the concentration of nitrite ions (NO_2_^−^) was monitored by recording the absorbance of aqueous solutions (after adding the Griess’ reagent) on a UV-Vis spectrophotometer (Hitachi U-2910, Tokyo, Japan). In this respect, 0.2 mL of Griess’ reagent was added to 4.0 mL of the analyzed aqueous solution. Next, the resulting solution was gently stirred for about 30 min at 27 °C to promote the complexation reaction between Griess’ reagent and nitrite ions. The absorbance of the aqueous solution was recorded at 525 nm wavelength and then converted to concentration using the linear regression equation resulting from the calibration curve (absorbance—concentration). The molar extinction coefficient from the calibration curve was equal to ε = 31,100 M^−1^ cm^−1^. It should be mentioned herein that in a classical Griess’ reaction, nitrite ion (NO_2_^−^) reacts with sulfanilic acid (HO_3_SC_6_H_4_—NH_2_) to generate a diazonium cation (HO_3_SC_6_H_4_—N^+^≡N), which then couples with α-naphthylamine (C_10_H_7_NH_2_) in para-position to form an azo dye of a red-violet color (HO_3_SC_6_H_4_—N = N—C_10_H_6_NH_2_) [3,21]. Additional details regarding Griess’ reaction are reported in Scheme S1 from the Supplementary Materials Section S1.

The fabricated composite membrane (in laboratory conditions) was characterized morphologically and structurally. In this regard, the surface examination was carried out by scanning electron microscopy (SEM) using an (ESCM) Quanta 200 device (Brno, Czech Republic) equipped with Energy Dispersive X-ray (EDX) module. The recorded SEM micrographs were further analyzed by means of ImageJ open-source software. Atomic force microscopy (AFM, NTEGRA Spectra NT-MDT, Zelenograd, Russia) was also involved to explore the roughness of the membrane surface. Infrared spectra with Fourier-transform (FTIR) were recorded in the range of 400–4000 cm^−1^ by using a Bruker Vertex 70 FTIR spectrometer (Ettlingen, Germany).

## 3. Computational Protocol

### 3.1. Multiple-Regression Modeling by Response Surface Methodology (RSM)

Response surface methodology (RSM) is a mathematical-statistical tool dealing with the multiple-regression modeling applied to real-world problems. In science and engineering, the RSM gained a wide application for exploring the functional relationship between one or more responses (output variables) and the factors (input variables) of a process or system [22,23]. In this respect, the RSM is often employed for empirical (or data-driven) modeling and implies the doing of experiments in accordance with an adopted design of experiments (DoE) [24]. Such a plan (DoE) involves conducting experiments by simultaneously varying the factors, leading to saving resources for experimentation if compared to the one-variable-at-a-time methodology. Consequently, the study by RSM involves four important aspects [22,23,24,25,26]: (1) experimental planning, (2) data analysis, (3) data-driven modeling, and (4) model-based optimization. To develop a reliable empirical model, the input variables (factors) are usually subjected to codification (see Supplementary Materials Section S2). The codification procedure is necessary to investigate in the same framework (factorial space) the input variables that are of different orders of magnitude in the real space [25,26]. Generally, the response surface model represents a second-order polynomial equation with interaction terms, which can be written compactly using the matrix-vector notation [22,23,24]:

(1) $${\hat{Y}}_{RSM}\left(x\right)=b_{0}+x^{T}b_{L}+x^{T}Bx$$

where ${\hat{Y}}_{RSM}$ denotes the estimated response by the RSM model; x—vector of input variables (factors), $x^{T}$—transpose of the vector x; $b_{0}$—intercept coefficient; $b_{L}$—vector of linear regression coefficients ($b_{i}$), and **B**—matrix of quadratic and interaction regression coefficients, i.e., with (*i*)th element equal to ($b_{ii}$), and the (*ij*)th off-diagonal element equal to (1/2 × $b_{ij}$).

Hence, the issue of empirical modeling is reduced to the calculation of the regression coefficients by means of the multiple-regression technique [25,26]:

(2) $$b={\left(X^{T}X\right)}^{-1}X^{T}y$$

where b is the column vector of the regression coefficients b = {$b_{0}$, $b_{i}$, $b_{ii}$, $b_{ij}$}^T^ that includes the main elements of $b_{0}$, $b_{L}$, and **B**; X—matrix of experimental input data (coded values of input variables), $X^{T}$—transpose matrix, and y—column vector of output experimental data (observed response). In this study, the RSM model was constructed by means of the Design-Expert 10 software program.

### 3.2. Machine Learning by Artificial Neural Network (ANN)

Artificial neural networks (ANNs) represent a subfield of machine learning theory. The development of the concept of modeling by artificial neural networks (ANNs) was inspired by information processing and distributed communication nodes that exist in biological systems. In the last 2.5 decades, artificial neural networks (ANNs) were successfully applied for modeling the separation and purification processes in membrane science [27]. An artificial neural network (ANN) is made of a set of computation units (artificial neurons) interconnected by synaptic connections and biases. Each connection has an associated numerical weight. The set of synaptic numerical weights ($w_{1}$, $w_{2}$, …, $w_{n}$) along with the biases (**θ**, *θ*) represents the numerical parameters of the artificial neural network.

An artificial neuron signifies a computational node that has several inputs and one output. Mathematically, the artificial neuron involves two functions [27,28,29,30]: (1) the summation function and (2) the activation function. The summation function allows the aggregation of input signals ($x_{i}$) into an integrated signal ($\lambda_{j}=\sum w_{ij}x_{i}+\theta_{j}$). Then, the integrated signal ($\lambda_{j}$) is taken over by the activation function, which generates the output signal of the neuron ($out_{j}$ = *f*($\lambda_{j}$)) and transfers it to the network (see Supplementary Materials Scheme S2) [31]. The most typical activation functions applied to solve multiple-regression problems are (1) linear function (*purelin*), (2) sigmoidal function (*logsig*), and (3) hyperbolic tangent (*tansig*) [27,28].

The placement, organization, and interconnection of artificial neurons in the network define the architecture (topology) of the network, which can be represented by a support graph. The network architecture involves several layers, namely: the inputs, the hidden layer (or hidden layers), and the output layer. The computation nodes (artificial neurons) are placed in hidden and output layers.

To address the multiple-regression problem by using the ANN concept, a multilayer neural network with unidirectional architecture (feed-forward) is most often applied. In a feed-forward ANN topology (also known as multi-layer perceptron MLP), the information flows in a single direction (input → output). For this type of unidirectional architecture, the support graph does not contain cycles (neurons are placed on consecutive levels), and the output vector is determined by direct calculation from the input vector. The estimation of the response function by a feed-forward ANN (with one hidden layer and one output layer) can be written by using the following vector-matrix expression:

(3) $${\hat{Y}}_{ANN}\left(x\right)=f^{\left(2\right)}\left(LW^{\left(2,1\right)}f^{\left(1\right)}\left(IW^{\left(1,1\right)}x+\theta^{\left(1\right)}\right)+\theta^{\left(2\right)}\right)$$

where ${\hat{Y}}_{ANN}$ denotes the network output (predictions given by ANN model), x is the vector of input variables (inputs), $f^{\left(1\right)}$—vector of the activation functions assigned to the hidden layer (layer-1), $f^{\left(2\right)}$—vector of the activation functions attributed to the output layer (layer-2), $IW^{\left(1,1\right)}$ = $w_{ij}{}^{\left(1,1\right)}$ is the input weight matrix, $LW^{\left(2,1\right)}$ = $w_{ij}{}^{\left(2,1\right)}$ is layer weight vector, $\theta^{\left(1\right)}$ = ${\left(\theta_{j}\right)}^{\left(1\right)}$—bias vector, and $\theta^{\left(2\right)}$ is the bias scalar. To develop a robust ANN model, a pre-processing step and a post-processing step are usually applied (see Supplementary Materials Section S3).

In the course of the machine learning procedure, the feed-forward artificial neural network (ANN) is trained by applying an algorithm involving the back-propagation of errors. This algorithm allows the adjustment of the parameters of the neural network (i.e., weights and biases) in order to minimize the residual error between the network output (${\hat{Y}}_{ANN}$) and the target experimental response ($Y_{target}$). Thus, the performance of training is typically expressed via the mean-square-error function $MSE\sim \sum {\left({\hat{Y}}_{ANN}-Y_{target}\right)}^{2}$. Consequently, the weights and biases are adjusted by means of recursive schemes (e.g., $w_{ij}^{new}=w_{ij}^{old}+\Delta w_{ij}$ and $\theta_{j}^{new}=\theta_{j}^{old}+\Delta \theta_{j})$ while minimizing the MSE function. One of the most efficient back-propagation algorithms is based on the Levenberg–Marquardt method [32,33]. In our study, the ANN model was developed using the standard neural network toolbox implemented in the Matlab program.

### 3.3. Machine Learning by Support Vector Machine (SVM)

By developing the machine learning domain, mathematicians and computer scientists have proposed another interesting technique known as the support vector machine (SVM), which can be employed for response function estimation. Hence, the support vector machine (SVM) represents a powerful methodology for solving problems in machine learning, such as function estimation by regression analysis, nonlinear classification, and pattern recognition [34,35,36,37,38,39,40,41,42,43]. In this study, the emphasis was put on the application of SVM for response function estimation by multiple regression. The basic idea of SVM and support vector regression (SVR) consists in using the kernel function $K\left(x,x_{\tau }\right)$, which is the dot product of two vectors in the feature space (i.e., superior space) defined by the mapping functions [34,36,41]:

(4) $$K\left(x,x_{\tau }\right)=\varphi {\left(x\right)}^{T}\varphi \left(x_{\tau }\right)$$

where x denotes the vector of input variables; $x_{\tau }$ is the vector of input variables associated with the training set; and $K\left(x,x_{\tau }\right)$ is the kernel function. Thus, the input vectors x and $x_{\tau }$ are mapped into a high-dimensional feature space defined by the transforming functions φ(x) and $\varphi \left(x_{\tau }\right)$. According to SVM methodology, there is no need to define explicitly the mapping functions φ(x) and $\varphi \left(x_{\tau }\right)$ since SVR requests only the dot product between vectors in the regression procedure. Hence, in the frame of SVR, the kernel function $K\left(x,x_{\tau }\right)$ operates to find a hyperplane in the higher-dimensional feature space (φ(x)) without increasing the computational cost. This hyperplane in feature space corresponds to a response surface of nonlinear type when we transform the hyperplane back to the original space of inputs.

The most popular kernel function employed in SVR is the Gaussian RBF kernel (radial basis function). The Gaussian RBF kernel is widely used in SVM owing to its flexibility; it represents the square of the Euclidean distance between the two vectors, that is [34,39,41]:

(5) $$K\left(x,x_{\tau }\right)=exp\left(\frac{-{\left(\left||x||-||x_{\tau }|\right|\right)}^{2}}{\sigma^{2}}\right)$$

where $\sigma^{2}$ denotes the squared bandwidth parameter for the Gaussian RBF kernel. Ultimately, the estimation of the response function by means of SVM can be written as [34,42]:

(6) $${\hat{Y}}_{SVM}\left(x\right)=\overset{n_{\tau }}{\underset{\tau =1}{\sum }}\alpha_{\tau }K\left(x,x_{\tau }\right)+\beta$$

where $\alpha_{\tau }$ and β are parameters of the SVM model determined by the regression procedure.

In this study, the least squares support vector machine (LS-SVM) was applied to address the function estimation problem by machine learning. Hence, we employed the LS-SVMlab Toolbox (v.1.5, Katholieke Universiteit Leuven, Leuven-Heverlee, Belgium) developed by Suykens and co-workers [43,44]. As designed, this toolbox was used in the frame of the commercial Matlab package. Additional details regarding SVM are given in the Supplementary Materials (Section S4).

## 4. Results and Discussions

### 4.1. Design of Experiments (DoE)

The adsorption-ultrafiltration experiments, designed to remove nitrite ions (NO_2_^−^) from aqueous solutions, were performed in accordance with a central composite experimental plan of rotatable type (Table 1). As feed solution, we considered water contaminated synthetically with nitrite ions in high concentration in all experiments, i.e., [NO_2_^−^]_0_ = 5 mg/L. This level exceeds the maximum allowed concentration of tenfold. The key factors considered as having the main influence on the performance of the adsorption-ultrafiltration process were (1) sorbent dose *SD* (% *w*/*v*) and (2) pH of feed solution. As sorbent, we employed the organoclay adsorbent (TmSA-MMT), representing the montmorillonite (MMT) clay modified with trimethyl stearyl ammonium TmSA (C_21_H_46_N^+^). The idea here was that the positively charged moiety of TmSA (C_21_H_46_N^+^) from organoclay can interact with negatively charged nitrite anions (NO_2_^−^) by electrostatic forces. This assumption was supported by molecular docking simulation reported in Supplementary Materials (Section S5). The region of experimentation was defined by the ranging intervals of the main factors, i.e., *SD* (0.12–0.58% *w*/*v*) and pH (4.9–9.1), as given in Table 1.

The ultrafiltration experiments were carried out in dead-end mode, and the experimental set-up is depicted in Figure 1. In a typical experiment, the organoclay adsorbent (TmSA-MMT) was added to the aqueous solution containing NO_2_^−^ ions, in accordance with the designed sorbent dosage (Table 1). Then, the pH of the aqueous solution was adjusted to the required value (Table 1), and the resulting feed solution was magnetically stirred for 60 min to attain equilibrium. This period of time was fixed to attain equilibrium in accordance with the kinetics of adsorption detailed in Supplementary Materials (Figure S1 and Table S1). It should be mentioned that the observed maximum adsorption capacity of the nanoclay (TmSA-MMT) for nitrite ions was found to be 2.57 mg/g, according to the adsorption isotherm of type III (see Supplementary Materials Figure S2). After attaining equilibrium, the colloidal suspension (TmSA-MMT/NO_2_^−^) was transferred to the UF stirred cell, where the ultrafiltration process was performed at room temperature (24 ± 2 °C) under three bars (operating pressure). To minimize the concentration polarization effect, the colloidal suspension was gently stirred during ultrafiltration. Thus, the separation process was performed by retaining the particles of the adsorbent loaded with nitrite ions (TmSA-MMT/NO_2_^−^) onto the membrane surface and collecting the purified water (permeate flux). The performance of separation of the hybrid process (adsorption-ultrafiltration) was evaluated by determining experimentally the removal (rejection) efficiency (*Y*, %) of nitrite ions, which can be expressed as:

(7) $$Y=\left(1-\frac{{\left[NO_{2}^{-}\right]}_{P}}{{\left[NO_{2}^{-}\right]}_{0}}\right)\times 100$$

where ${\left[NO_{2}^{-}\right]}_{0}$ denotes the initial concentration of nitrite in feed solution (5 mg/L), and ${\left[NO_{2}^{-}\right]}_{P}$ is the concentration of nitrite in permeate solution (mg/L).

Table 1 reports the experimental conditions of the adsorption-ultrafiltration process as well as the main response (rejection efficiency *Y*, %) determined for each run. In addition, the initial permeate flux *J_i_* (recorded in the first three minutes of ultrafiltration) was determined experimentally. For the conditions given in Table 1, the initial permeate flux *J_i_* varied into the narrow interval of 176–189 (LMH, L∙m^−2^∙h^−1^) with an average value of 183 ± 5 (L∙m^−2^∙h^−1^).

### 4.2. Data-Driven Modeling: RSM vs. Machine Learning (ANN and SVM)

The experimental design reported in Table 1 represents the main matrix of data that was used to develop all three data-driven models (i.e., RSM, ANN, and SVM).

First, starting from the experimental data summarized in Table 1 and using the multiple-regression technique [25,26], we developed the RSM model that can be expressed in terms of coded variables as follows:

(8) $${\hat{Y}}_{RSM}=80.97+10.69x_{1}+1.93x_{2}-4.38x_{1}^{2}-5.06x_{2}^{2}-4.07x_{1}x_{2}$$

subjected to: $-\mathrm{ubjec}\leq x_{j}\leq 1.414;\left(j=1,2\right)$. 

After applying the mathematical substitution technique, the RSM model with actual factors can be written as

(9) $${\hat{Y}}_{RSM}=-115.04+235.92\times SD+38.18\times \mathrm{pH}-109.47\times SD^{2}\;-2.25\times {\mathrm{pH}}^{2}-13.56\times SD\times \mathrm{pH}$$

subjected to: 0.12≤SD≤0.68(%w/v);4.9≤pH≤9.1.

**Figure 2:**
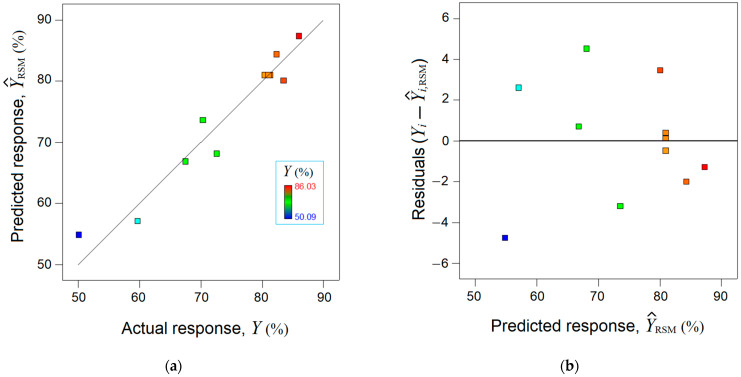
Agreement between experimental data and RSM model: (**a**) Predicted response by RSM vs. actual response, ($r_{LC}^{2}$ = 0.939); (**b**) residual errors against predicted response $\left({\hat{Y}}_{RSM}\right)$.

The mathematical RSM model was validated statistically by using the analysis of variance (ANOVA), which is detailed in Supplementary Materials (Section S6). In addition, the accordance between the observed response (Y, %) and the estimated response by the RSM model $\left({\hat{Y}}_{RSM}\right)$ was analyzed (Figure 2). The parity plot between the actual response and the predicted one disclosed a reasonable alignment of data along the bisector, indicating a linear correlation coefficient equal to $r_{LC}^{2}$ = 0.939 (Figure 2a). Figure 2b shows the residual errors (between experimental data and RSM model) against predicted response, suggesting a normal distribution of the residuals.

**Figure 3:**
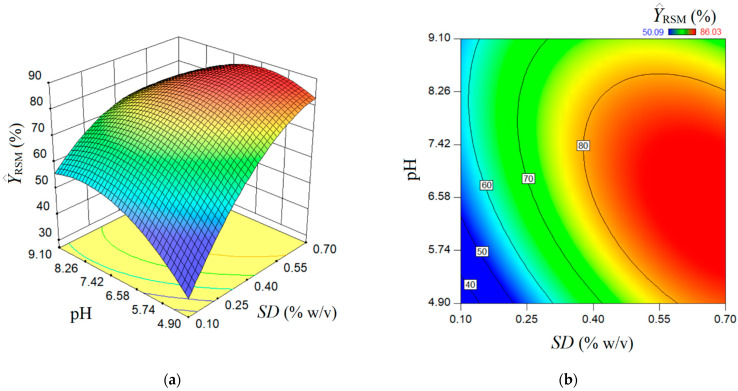
Predictions given by RSM model: (**a**) response surface plot (3D graph), and (**b**) contour-lines map (2D) showing the estimated response $\left({\hat{Y}}_{RSM}\right)$ depending on pH and *SD* (g/L) factors.

The estimated response surface by the conventional multiple-regression model (RSM) is depicted in Figure 3, showing a surface of maximum type with some ridge aspects. Herein, the 3D graph (Figure 3a) and 2D contour-line map (Figure 3b) show the reciprocal influence of *SD* and pH factors on the estimated rejection efficiency $\left({\hat{Y}}_{RSM}\right)$. In accordance with the prediction given by the RSM model, the main effect of the *SD* factor (sorbent dose) is greater than the main effect of the pH factor. Generally, the greater the *SD* factor is, the higher the estimated response $\left({\hat{Y}}_{RSM}\right)$. Likewise, an interaction effect exists between the factors *SD* and pH. According to this interaction effect, the increment of pH value at a low sorbent dose conducts gentle increases of the rejection efficiency $\left({\hat{Y}}_{RSM}\right)$. At a high adsorbent dose, the increment of pH value from 4.9 to 8.0 does not affect the response too much; for pH > 8.0, the rejection efficiency is gently diminished. The response surface plot shown in Figure 3 indicates an optimal region (red-colored zone), where the estimated response attains elevated values $({\hat{Y}}_{RSM}$ > 80%). In the first approximation by visual analysis (Figure 3), this optimal region can be characterized in terms of factors as follows: *SD* > 0.55% *w*/*v* and pH 5.3–8.3. More precise localization of the optimal point by RSM is detailed in the next section, where the numerical optimization is discussed.

Second, regarding machine learning by using the artificial neural network (ANN), the experimental plan reported in Table 1 served as the main matrix of data used for ANN training. To improve the robustness of the neural network, additional sets of data (validation and test) were also employed for the development of the ANN model. These validation and test sets of data are reported in Supplementary Materials (Section S3, Table S2).

**Figure 4:**
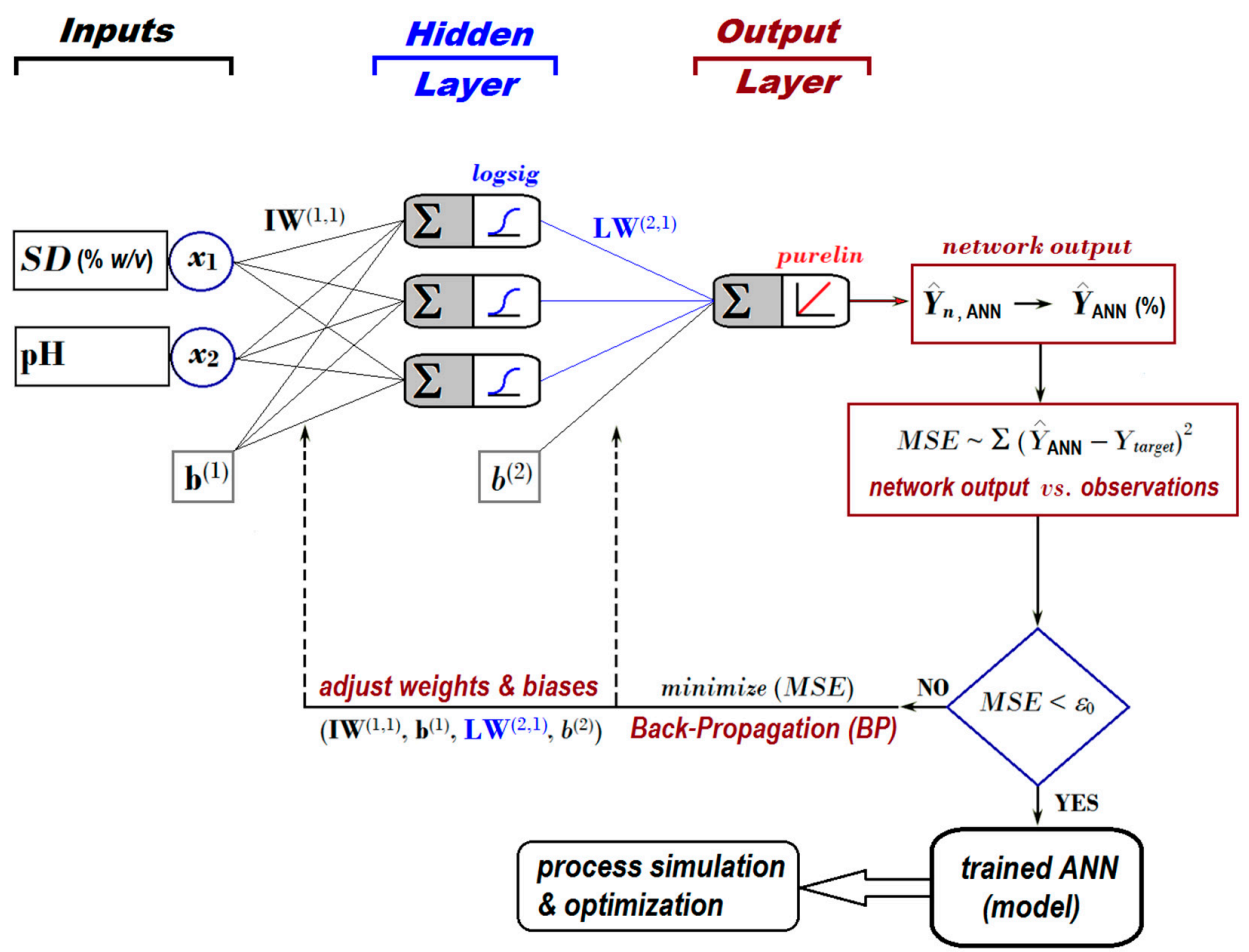
Feed-forward ANN architecture [2:3:1] developed to predict the removal efficiency of nitrite ions from aqueous solutions by using the adsorption-ultrafiltration hybrid process.

For this application, we employed a feed-forward ANN, whose architecture is depicted in Figure 4. The topology of the applied neural network included (1) two inputs (associated with factors *SD* and pH); (2) one hidden layer with three artificial neurons activated by logsig nonlinear function; and (3) one output layer with a single artificial neuron activated by purelin linear function (Figure 4).

**Figure 5:**
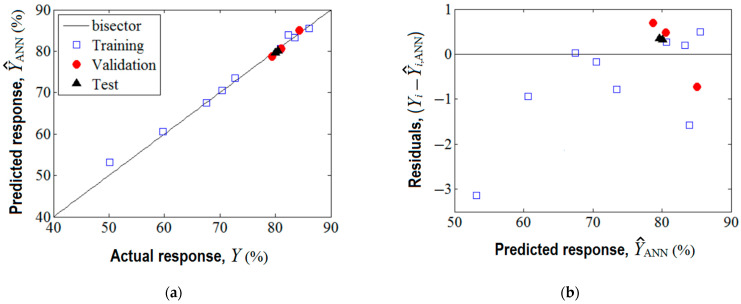
Agreement between experimental data and ANN model: (**a**) ANN output (predicted response) vs. actual response (target), ($r_{LC}^{2}$ = 0.994); (**b**) residual analysis: residual errors against predicted response $\left({\hat{Y}}_{ANN}\right)$.

The training of this network was realized by means of Levenberg–Marquardt back-propagation algorithm. The training performance was achieved in four epochs (see Supplementary Materials Figure S3), and the established optimal network parameters (i.e., weights and biases) are reported in Supplementary Materials (Table S3). The agreement between experimental data (target) and the predicted response given by the ANN model is shown in Figure 5. As one can see from Figure 5a, the data from training, validation, and test sets are aligned along the bisector revealing a linear correlation coefficient of $r_{LC}^{2}$ = 0.994. This proves good prediction ability of the developed ANN model.

**Figure 6:**
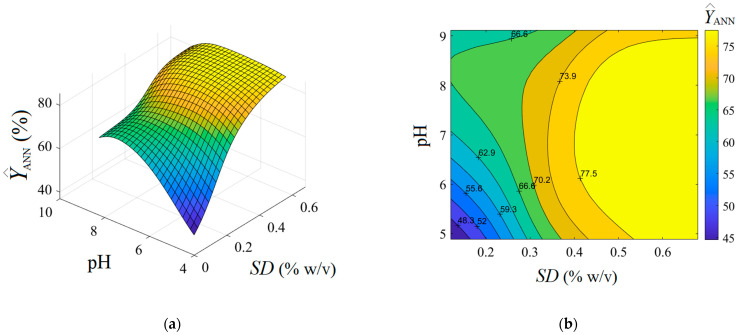
Predictions given by feed forward ANN model showing the estimated response $\left({\hat{Y}}_{ANN}\right)$ depending on pH and *SD* (g/L) factors: (**a**) 3D output surface, and (**b**) 2D contour-lines map.

Optionally, the analysis of variance (ANOVA) was performed highlighting the significance of the ANN model (see Supplementary Materials Section S6). As detailed in Figure 5b, the residual errors (between observations and the ANN model) are of both negative and positive values. The generalization capability of the developed ANN model is displayed in Figure 6, where the predictions provided by the neural network are plotted as a 3D output surface (Figure 6a) and a 2D contour-lines map (Figure 6b). The response surface provided by the ANN model (Figure 6) is somewhat similar to the one generated by the RSM model (Figure 3), excepting some specific differences. The similitude is related to the general shape of the ANN output surface for the region of experimentation, that is, the tendency to a maximum shape with slight ridge aspects (Figure 6). Instead, the discrepancies imply the following: The optimal region indicated by ANN (yellow-colored zone in Figure 6) seems to be more extended, i.e., this optimal region might be defined by *SD* > 0.5% *w*/*v* and pH 5.3–8.7; Moreover, an inflection point of the ANN output surface appears at *SD* 0.24–0.26% *w*/*v* and pH 8.2.–8.4 (Figure 6), which does not emerge for the surface estimated by RSM. This might be explained by the ability of the ANN model to better estimate more complex nonlinear effects compared to the RSM model.

**Figure 7:**
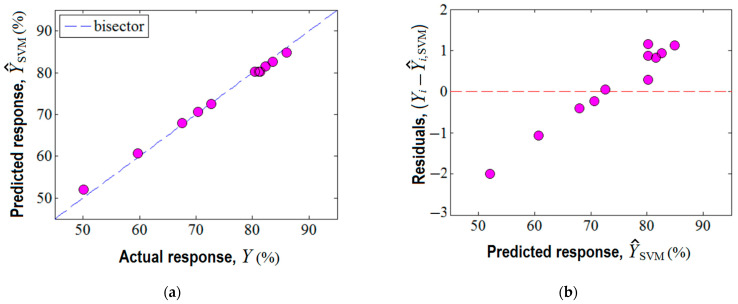
Agreement between experimental data and SVM model: (**a**) predicted response by SVM vs. actual response, ($r_{LC}^{2}$ = 0.999); (**b**) residual errors against predicted response $\left({\hat{Y}}_{SVM}\right)$.

Third, the machine learning technique based on the support vector machine (SVM) was also employed for building a predictive model for the same region of experimentation. In this respect, the experimental design given in Table 1 served as the matrix of data to develop the SVM model. The established parameters for the SVM model are reported in Supplementary Materials (Table S4). The prediction ability of the SVM model is displayed in Figure 7. Herein, the accordance between the experimental results and the SVM model is shown (Figure 7a), revealing an excellent alignment of data near the bisector with a linear correlation coefficient of $r_{LC}^{2}$ = 0.999. This fact suggests good prediction ability of the constructed SVM model. In addition, the analysis of variance (ANOVA) corroborated the statistical significance of the SVM model (Supplementary Materials Section S6). The plot of residuals against the predicted response by SVM is highlighted in Figure 7b, indicating the existence of both negative and positive values of the residual errors.

**Figure 8:**
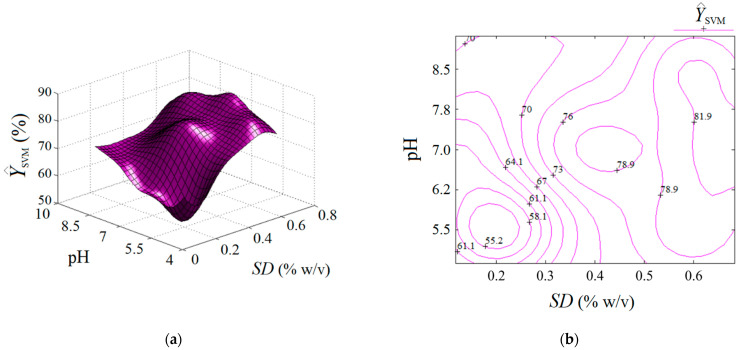
Predictions given by SVM model: (**a**) response surface plot (3D graph), and (**b**) contour-lines map (2D) showing the estimated response $\left({\hat{Y}}_{SVM}\right)$ depending on pH and *SD* (g/L) factors.

The estimated response by the SVM model is represented in Figure 8 as an output surface influenced by *SD* and pH factors. Predictions estimated by the SVM model are also plotted in both formats, i.e., 3D graph (Figure 8a) and 2D map (Figure 8b). Compared to the previous two cases (RSM and ANN), the output surface provided by SVM shows several extreme points inside the valid region, i.e., (1) a local minimum (at *SD* ≈ 0.2% *w*/*v*, pH 5.5); (2) the first local maximum (at *SD* ≈ 0.43% *w*/*v*, pH 7.2); (3) the second local maximum (defined by the region *SD* > 0.65% *w*/*v*, pH 6.2–7.8). Hence, the SVM model showed a different ability to describe the nonlinear effects than the ANN and RSM models, providing a more complex response surface with more than one extreme point inside the region of experimentation. This might be attributed to the radial distribution function (RDF) implied in the SVM algorithm.

The built data-driven models (RSM, ANN, and SVM) offer functional relationships between the input and output variables of the process, without giving insights into the interaction mechanism at the molecular level. To address this aspect, we employed molecular docking simulations, which revealed the importance of the electrostatic interactions in the retention of nitrite ions, as detailed in Supplementary Materials (Figure S4 and Table S5) [44,45].

All three employed models (RSM, ANN, and SVM) were significant from a statistical viewpoint, as suggested by the analysis of variances (see Supplementary Materials Tables S6–S8). Likewise, all three models were tested for normal distribution of residual errors (for a confidence interval of 95%), and the results were reported as probability plots (Supplementary Materials Figure S5a–c). According to these probability plots, the best normal distribution of residuals was observed for the RSM model followed by the SVM model. In the case of the ANN model, a slight deviation from the normal distribution was observed for a single point (Supplementary Materials Figure S5b). Table 2 summarizes the residual analysis and descriptive statistics of residual errors aiming to compare the prediction abilities of the constructed models RSM, ANN, and SVM. According to descriptive statistics, the amplitude represents the difference between the maximal residual error and the minimal residual error. In Table 2, the lowest amplitude (3.1567) is attributed to the SVM model, followed by the ANN model which gives a slightly higher amplitude (3.8252). The amplitude associated with the RSM model is almost threefold higher, i.e., (9.2720). Likewise, the statistical parameters like linear correlation coefficient ($r_{LC}^{2})$ and ANOVA coefficient of determination (*R^2^*) were found to be the best for the SVM model, followed by the ANN model, and then by the RSM model. Thus, according to the residual analysis, the prediction performance of the models slightly diminished in the following order: SVM > ANN > RSM. The superiority of SVM over ANN in response estimation with lower residual errors might be attributed to the fact that SVM possesses a high generalization capability in solving practical problems related to nonlinearity, small samples, and over-fitting [39]. The residual analysis performed for this case study suggested that the response surface predicted by the SVM model might be closer to the true response surface, if compared to the ANN and RSM models.

### 4.3. Multivariate Optimization of Adsorption-Ultrafiltration Hybrid Process

In this study, the multivariate optimization problem dealt with the maximization of the response function (objective function), representing the rejection efficiency depending on the two variables *x*_1_ and *x*_2_ (that correspond to *SD* and pH factors). Hence, each response function estimated by RSM, ANN, and SVM models was subjected to maximization in the optimization problem. To this end, the Monte Carlo optimization method was employed, which relies on stochastic search using the pseudorandom number (PRN) generator [46,47]. Pseudorandom numbers generated by computers are usually distributed uniformly between 0 and 1.

The computer-aided numerical optimization was performed by generating pseudorandom numbers (in the interval 0–1) that were used to generate the vector of input variables according to the following equation:

(10) $$x^{\left(k\right)}=\left\{\begin{array}{c} x_{1}^{\left(k\right)}\\ x_{2}^{\left(k\right)} \end{array}\right\}=\left\{\begin{array}{c} L_{1}+{\mathrm{PRN}}_{1}^{\left(k\right)}\left(U_{1}-L_{1}\right)\\ L_{2}+{\mathrm{PRN}}_{2}^{\left(k\right)}\left(U_{2}-L_{2}\right) \end{array}\right\}$$

where $x^{\left(k\right)}={\left[x_{1}^{\left(k\right)},x_{2}^{\left(k\right)}\right]}^{T}$ denotes the vector of input variables (in coded values); *k* is the iteration number associated with the random number generation loop (*k* ∈ *K*; *K* = 10^6^); $L_{i}$ and $U_{i}$ are the lower and upper bounds of the input variables (i.e., $L_{i}$ = −1.414 and $U_{i}$ = +1.414), respectively, and ${\mathrm{PRN}}_{i}^{\left(k\right)}$ represents the pseudorandom number generated for the variable (*i*) at iteration (*k*). Thus, the recursive Equation (10) enabled the conversion of the pseudorandom numbers (PRNs) into values of input variables uniformly distributed inside the valid region (i.e., the region of experimentation). Then, the response ($\hat{Y}$) was estimated by means of the model (RSM, ANN, or SVM) for each generated vector $x^{\left(k\right)}$. Finally, the calculated values of the response function $\hat{Y}(x^{\left(k\right)}$) were compared, and the maximal value was identified for each set and retained as the optimal solution. Table 3 reports the optimal points provided by each considered model (RSM, ANN, and SVM). These optimal conditions established by numerical optimization using the Monte Carlo method were checked experimentally in so-called confirmation runs. Generally, all three optimal solutions indicated by different models (RSM, ANN, and SVM) converged to the same region defined by sorbent dose 0.67–0.68% *w*/*v* and pH 6.4–7.1. In this optimal region, the observed response was about 86%, according to confirmation runs (Table 3). However, speaking more precisely, the optimal conditions (*SD* = 0.674% *w*/*v* and pH 7.0) provided by the SVM model were the best, since the highest experimental response of 86.28% for removal of nitrite ions was observed. By comparing the observed response (confirmation runs) given in Table 3, our assumption that the estimated SVM response surface is closer to the true response surface was corroborated. The observed value of 86.28% for rejection efficiency (under conditions indicated by the SVM model) was the highest one in all experiments where the commercial flat-sheet membrane (Ultracel^®^ 10 kDa) was used (see Table 1 and Table 3). It should also be mentioned that the initial permeate flux determined for Ultracel^®^ membrane under the optimal conditions given by SVM was equal to 185 ± 4 LMH (L∙m^−2^∙h^−1^). In the next section, a new composite porous membrane was tested under optimal conditions indicated by the SVM model.

### 4.4. Testing Optimal Conditions on a New Composite Membrane

The aim of the study presented in this section was to test a more retentive porous membrane for the removal of nitrite ions under the optimal conditions established previously by the SVM model (i.e., *SD* = 0.674% *w*/*v* and pH 7.0). The more retentive porous membrane was prepared in laboratory conditions using the phase-inversion method [48,49,50,51,52]. In this respect, the non-reactive thermoplastic co-polymer PVDF-HFP was used as the primary polymeric material for membrane fabrication. In addition, the halloysite (HS) nanotubes were used as inorganic fillers to add strength reinforcement to the composite membrane and induce hydrophilic properties. As a pore-generator agent (porogen), polyethylene glycol (PEG-400) was employed. As the aprotic solvent, for preparing casting solution, N, N-Dimethylacetamide (DMAc) was used. The resulting casting solution (PVDF-HFP/HS/PEG/DMAc) was then poured on a glass plate and stretched to a thin layer of 300 um thickness, using a film applicator (ZUA 2000, ZEHNTNER). Then, the glass plate was immersed in distilled water to promote the phase-inversion process and membrane formation. More details regarding the synthesis of the composite membrane by the phase-inversion method are reported in Supplementary Materials (Section S7). This composite membrane was designed to have a final composition (in the solid phase) of 97% PVDF-HFP and 3% HS (halloysite) by weights. The thickness of the resulting membrane (PVDF-HFP/HS) was determined by micrometer measurements and it was 112 ± 18 μm.

**Figure 9:**
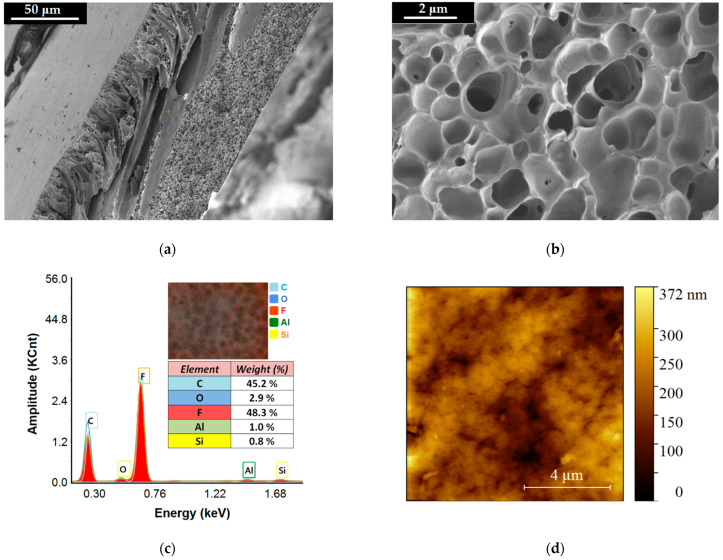
Morphological and structural characteristics of the produced composite membrane PVDF-HFP/HS: (**a**,**b**) SEM images (cross-section) of the composite membrane recorded at different magnitudes; (**c**) EDX spectrum for the composite membrane (inset image—distribution map of chemical elements); (**d**) AFM scanning-map (2D) of the top surface of the composite membrane (PVDF-HFP/HS).

The fabricated composite membrane (PVDF-HFP/HS) was characterized by physical-chemical instrumental techniques (i.e., SEM-EDX, FTIR, AFM, and WCA). Figure 9 highlights the morphological and structural features of the produced composite membrane. The microscopic images (SEM) at different magnitudes are given in Figure 9a,b, where the porous morphology of the composite membrane is detailed in cross-section. Additional SEM micrographs are reported in Supplementary Materials (Figure S6).

The careful inspection of the registered SEM micrographs enabled us to discern three layers (in the membrane cross-section) with different patterns of pores. The first one (Layer-1, L1) represents the top skin layer with a thickness of 5.80 ± 1.75 μm and involves small pores (0.05–1.98 μm) that ensure selective separation. That is, the top layer L1 contributes to the retention of the organoclay particles loaded with nitrite ions while enabling the permeation of water. The second layer (Layer-2, L2) is of thickness 60.72 ± 17.62 μm and implies the finger-like macropores (3.98–34.50 μm) as well as the macrovoids (>35 μm). This layer of macropores (L2) contributes to the hydrodynamic conditions of ultrafiltration by improving the flow of the permeate flux. The third layer (Layer-3, L3) is the bottom layer of thickness 46.42 ± 13.97 μm, comprising the sponge-like pores (0.10–2.76 μm), which are illustrated in Figure 9b. These sponges-like pores have the role of maintaining a good permeate flow as well as sustaining the stability of the bottom scaffold (L3) of the membrane. In addition, the statistical analyses of the pore size distributions were performed for each considered layer. In this respect, the histograms of pore size distributions were built (see Supplementary Materials Figures S7–S9). The analysis of the constructed histograms showed distributions skewed to the right, suggesting lognormal distributions (Figures S7–S9).

**Figure 10:**
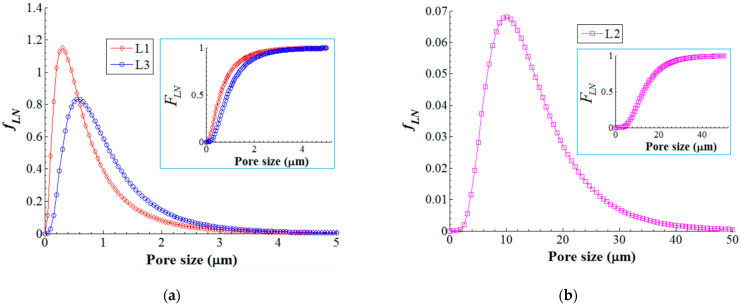
Lognormal probability density function (*f*_LN_) of the pore size distribution for composite membrane (PVDF-HFP/HS): (**a**) pore size distribution in layers L1 and L3; (**b**) pore size distribution in layer L2; inset images—cumulative distribution function (*F*_LN_).

Therefore, on the basis of histograms, we developed the lognormal probability density function (*f*_LN_) as well as the corresponding cumulative distribution function (*F*_LN_) that is shown in Figure 10. As one can see from Figure 10a,b, the probability density function (*f*_LN_) is comparable for the layers L1 (skin layer pores with mode 0.30 μm) and L3 (sponge-like pores with mode 0.58 μm), and obviously different for the layer L2 (finger-like pores with mode 10.0 μm). Moreover, the cumulative density function (insets in Figure 10a,b) revealed that (1) 75% of accounted pores were less than 1.0 μm in size for L1 (skin layer); (2) 75% of accounted pores were less than 18.3 μm in size for L2 (finger-like structure); and (3) 75% of accounted pores were less than 1.5 μm in size for L3 (sponge-like structure).

Likewise, the distribution of the pore sizes from the top surface of the membrane was performed based on the analysis of SEM images. The constructed histogram of the pore size distribution for these opening pores is detailed in Figure S10 from Supplementary Materials. This histogram pointed out a relevant skewness for the distribution of opening pores ranging from 0.01 μm to 1.25 μm, disclosing a mode of 0.04 μm.

The overall porosity of the composite membrane (PVDF-HFP/HS) was determined gravimetrically by immersion of this material in isopropyl alcohol, and by measuring the weight of liquid retained in the pores [53,54] (for more details see Supplementary Materials Section S7). Thus, the overall porosity of the composite membrane (PVDF-HFP/HS) was found to be ε = 75.32 ± 2.65%, which was somewhat greater than the porosity (71.94 ± 3.04%) determined for the pristine polymeric membrane (PVDF-HFP).

According to the EDX spectrum reported in Figure 9c, the presence of all expected chemical elements (C, O, F, Al, Si) was proved. These emerged from two sources (1) PVDF-HFP polymer and (2) halloysite nanotubes (HS). The EDX spectrum for the composite membrane (Figure 9c) pointed out a much lower weight percentage (0.8–2.9%) for Si, Al, and O from HS compared to the dominant elements C and F (45% and 48%) originating from polymer. This is due to the fact that HS was designed in a low amount (3% wt.) in the composite membrane, compared to the polymer matrix (97% wt.). Figure 9d illustrates the AFM scanning map (2D) of the top surface of the composite membrane (PVDF-HFP/HS). Results revealed that the surface of the membrane was not smooth but implied roughness in the form of nanosized hills and valleys. The average roughness parameter for the fabricated membrane (PVDF-HFP/HS) was found to be *R_a_* = 41.32 nm. The hills and valleys are shown in Figure 9d as bright and dark spots, respectively. From another perspective, the valleys (dark spots) from the AFM map might represent the openings or access pores to the membrane. The measured size of the dark spots from the AFM map ranged from 0.02 μm to 0.76 μm. This interval (0.02–0.76 μm) was framed into the wider interval of opening pores (0.01–1.25 μm) determined from the top surface of the membrane by SEM analysis (Supplementary Materials Figure S10).

It should be noted that the presence of the inorganic component HS (halloysite) in the polymeric matrix (PVDF-HFP) induced the hydrophilic properties to the composite membrane. This evidence was observed by measuring the water contact angle (WCA), which decreased from 80 ± 4° (for PVDF-HFP) to 61 ± 3° (for PVDF-HFP/HS), (see Supplementary Materials Figure S11).

**Figure 11:**
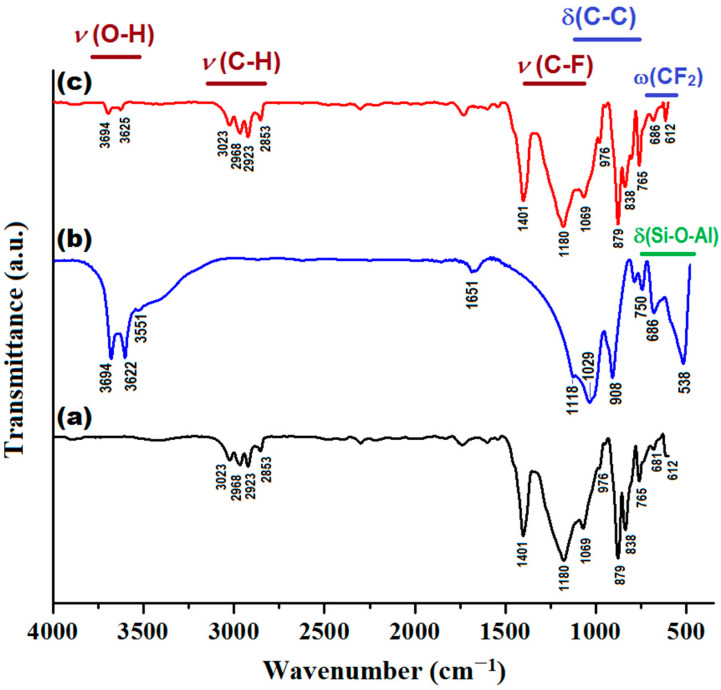
Infrared spectra (FTIR) of investigated materials: (**a**) PVDF-HFP membrane; (**b**) HS (Halloysite); (**c**) PVDF-HFP/HS composite membrane.

In addition, the produced composite membrane (PVDF-HFP/HS) was characterized by Fourier transform infrared spectroscopy (FTIR) and compared with the sample of neat co-polymer (PVDF-HFP) and halloysite (HS), as shown in Figure 11. Details regarding the assignments of the peaks from infrared (IR) spectra are given in Supplementary Materials (Section S8).

As shown in Figure 11, the IR spectrum of the composite membrane (PVDF-HFP/HS) is generally similar to the IR spectrum of the polymer (PVDF-HFP), since the weight of the polymer in the composite is significant (97%), while the weight of the inorganic component (HS) is small (only 3%). However, some changes can be distinguished in the IR spectrum of the composite compared to the pristine co-polymer. For instance, owing to the addition of HS, the peaks at 3694 and 3625 cm^−1^ (attributed to υ(O-H) stretching vibration from HS) can be clearly identified. In addition, for the composite membrane, the intensity of the peaks at 765 cm^−1^ and 686 cm^−1^ increased, to some extent, as a result of superimposing bending (δ) and wagging (ω) vibrations. That is, the overlay of the δ(Si-O-Al) vibration mode (from HS) to the vibration modes δ(C-C) and ω(CF2) of the polymer.

**Figure 12:**
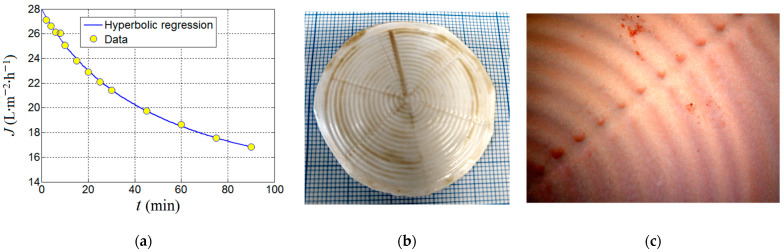
Outcomes of the ultrafiltration test performed by using PVDF-HFP/HS (3%) composite membrane: (**a**) permeate flux decline observed under optimal conditions (*SD* = 0.674% *w*/*v*, pH 7.0); (**b**) photo image of the spent membrane at the end of UF test; (**c**) the microscopic image that shows the top surface of the spent membrane highlighting the deposition of the cake fragments (distinguished as brown spots).

Finally, the produced composite membrane (PVDF-HFP/HS) was tested in a longer-term ultrafiltration assay (up to 90 min) under the established optimal conditions of adsorption-ultrafiltration (i.e., *SD* = 0.674% *w*/*v* and pH 7.0) in order to evaluate the kinetics of the permeate flux and the nitrite removal efficiency. Results from this ultrafiltration test aiming to remove nitrite ions by adsorption onto the organoclay are highlighted in Figure 12.

As one can see from Figure 12a, the permeate flux decreases against time. By assuming that the permeate flux is inversely proportional to time and may attain a steady-state level at equilibrium, the experimental data from Figure 12a were interpolated to a hyperbolic regression equation with three parameters that can be written as

(11) $$J\left(t\right)=\frac{J_{D}\times \tau }{\tau +t}+J_{E}$$

where *t* is the time of ultrafiltration (independent variable), and *J_D_*, *J_E_* and *τ* are the parameters of the adopted hyperbolic regression model. The model parameters *J_D_* and *J_E_* have the same units as permeate flux (e.g., LMH). The first parameter (*J_D_*) can be associated with the portion of the permeate flux subjected to the decline dynamics; thereby, it is denominated herein as the decline parameter (*J_D_*). The second parameter (*J_E_*) can be attributed to the portion of permeate flux that achieved a steady state; thereby, it is designated herein as the equilibrium or residual parameter (*J_E_*). The last parameter *τ* might be associated with the half-life of decline indicating the time when the declining term in Equation (11) is reduced by half (i.e., ½ *J_D_*). Thus, the parameter *τ* has the same units as time (e.g., min), and when *τ* = *t*, then the permeate flux is readily estimated as ½*J_D_* + *J_E_*. The interesting aspect of this hyperbolic model is that at time zero (*t* = 0), the initial permeate flux (*J_0_*) of the feed solution can be extrapolated to *J_0_* = *J_D_* + *J_E_*. For instance, in our case study (see Figure 12a), the parameters of the hyperbolic model were determined by nonlinear regression and were found to be *J_D_* = 17.3 LMH, *J_E_* = 10.7 LMH, and *τ* = 50 min. Consequently, the initial permeate flux (*J_0_*, at time zero *t* = 0) of the feed solution (containing [NO_2_^−^]_0_ = 5 mg/L) was estimated as *J_0_* = 28.0 LMH. This value was somewhat lower than the pure water flux (PWF_0_ = 31.1 ± 1.5 LMH) determined for the composite membrane. According to Figure 12a, in the course of 90 min (1.5 h) of filtration, the permeate flux was diminished but still had not completely achieved its steady state.

The meaning of the model parameters (*J_D_*, *J_E_*, and *τ*) can be distinguished from Figure S12 (given in Supplementary Materials), where the computer-aided simulation was performed based on the hyperbolic model for a larger time span. According to this plot, when filtration time is less than the half-life parameter (*t* < *τ*) the permeate flux decline is very fast, and afterward (for *t* > *τ*) the dynamics of the flux slows gradually until it reaches steady state at a permeate flux equal to *J_E_*.

It is worth mentioning here that the nitrite removal efficiency by the more retentive membrane (PVDF-HFP/HS) was equal to 95.0%, which represents a greater value than the one recorded for the commercial membrane (~86%). Note that a separation yield of 95% is a significant result. For a feed solution of [NO_2_^−^]_0_ = 5 mg/L (exceeding the maximum allowed value of ten times), the removal efficiency of 95% means about 0.25 mg/L of remaining nitrite ions are in the aqueous solution. This value is two times below the maximum allowed concentration. By comparing the performance of the commercial membrane (*Y*~86%, *J_0_* = 185 LMH) and the produced composite membrane (*Y* = 95%, *J_0_* = 28 LMH), it is evident that establishing the trade-off between the rejection efficiency and the permeate flux is essential for real applications. In our study, the focus was placed on the environmental protection aspect (i.e., removal of very toxic ions); thereby, the enhanced rejection was preferred instead of productive water flux. In this respect, the produced composite membrane (PVDF-HFP/HS) was more suitable for achieving this objective. It is important to note that the pristine montmorillonite (MMT) was also tested (in optimal conditions), disclosing a low rejection efficiency of 22.1%, compared to the rejection of 95.0% indicated by the organoclay (MMT-TmSA). Hence, the major role in the retention of nitrite ions can be attributed to the functional groups TmSA (C_21_H_46_N^+^) from the organoclay.

In Figure 12a, the permeate flux decline can be a consequence of the combined effect of concentration-polarization, membrane fouling, and membrane compaction. At the end of ultrafiltration, cake formation was noticed at the surface of the membrane (Figure 12b,c). The photo image of the spent membrane is shown in Figure 12b. In turn, Figure 12c displays the microscopic image unveiling the top surface of the spent membrane where the reddish spots (cake fragments) were deposited non-uniformly. The spent UF membrane was restored by rinsing it with tap water, then immersing it in 0.01 M NaOH solution for 1 min, and ultimately thoroughly washed with distilled water.

## 5. Conclusions

In summary, we demonstrated the capability of organoclay (Nanomer^®^ I.28E) to adsorb the nitrite ions (NO_2_^−^) and the efficiency of ultrafiltration to retain the formed colloids in a one-step hybrid process. The proposed mechanism of adsorption envisaged the interaction between organic moiety (C_21_H_46_N^+^) from organoclay and nitrite ion (NO_2_^−^). This interaction relied on electrostatic forces (Coulomb) as molecular docking simulation suggested. The UF experiments were conducted using a commercial porous membrane (Ultracel^®^) of 10 kDa MWCO. The hybrid adsorption-ultrafiltration process was modeled mathematically using three different computational tools, for comparison. Thus, the classical RSM modeling tool was compared with machine learning tools such as ANN and SVM. The residual analysis disclosed that machine learning by the SVM model provided the most accurate predictions compared to ANN and RSM models. For this case study, we believe that the true response surface was better estimated by the SVM model compared to the ANN and RSM models. The optimal conditions for the adsorption-ultrafiltration hybrid process were established by numerical optimization, disclosing an adsorbent dose of 0.674% *w*/*v* and pH 7.0. Under these optimal conditions (indicated by the SVM model), the nitrite removal efficiency was found to be 86.28% and the initial permeate flux was 185 LMH on the Ultracel^®^ commercial membrane. In addition, these optimal conditions of adsorption-ultrafiltration were tested on a more retentive membrane prepared in the laboratory by the phase-inversion method. This new composite membrane with 75.32% porosity (made of PVDF-HFP polymer and Halloysite (HS) inorganic nanotubes) revealed a higher nitrite removal efficiency of 95.0%, but a lower initial permeate flux of 28.0 LMH. Moreover, the kinetics of permeate flux decline was modeled by using a hyperbolic regression equation with three parameters. This hyperbolic regression model enabled the extrapolation of the permeate flux at time zero (*t* = 0).

## Figures and Tables

**Table 1:** Central composite experimental design of rotatable type employed for exploring the adsorption-ultrafiltration hybrid process for nitrite removal from aqueous solutions; fixed conditions for experimentations: [NO_2_^−^]_0_ = 5 mg/L; ΔP = 3 bar; temperature T = 298 K (25 °C).

Run/Trial	Sorbent Dose		pH of Feed Aqueous Solutions		Response: Nitrite Removal Efficiency *Y* (%)
	Coded *x*_1_	Actual *SD*, % *w*/*v*	Coded *x*_2_	Actual pH	
1	−1	0.20	−1	5.5	50.09
2	+1	0.60	−1	5.5	82.35
3	−1	0.20	+1	8.5	67.54
4	+1	0.60	+1	8.5	83.53
5	−1.414	0.12	0	7.0	59.69
6	+1.414	0.68	0	7.0	86.03
7	0	0.40	−1.414	4.9	72.64
8	0	0.40	+1.414	9.1	70.36
9	0	0.40	0	7.0	80.48
10	0	0.40	0	7.0	81.35
11	0	0.40	0	7.0	81.07

**Table 2:** Residual analysis and descriptive statistics of residual errors.

Statistical Descriptor for Residuals	Sources of Residuals (*Y_experimenta_*_l_ − *Y_model_*)		
	RSM Model	ANN Model	SVM Model
Minimal residue (min)	−4.7580	−3.1324	−2.0045
Maximal residue (max)	4.5140	0.6928	1.1522
Amplitude (max-min)	9.2720	3.8252	3.1567
Median	0.1030	0.1090	0.2822
Average	0.0002	0.3204	0.0672
Standard deviation	2.8035	1.0462	1.0079
$r_{LC}^{2}$ (LCC)	0.939	0.994	0.999
*R*^2^ (ANOVA)	0.938	0.989	0.992

**Table 3:** Optimal points established by model-based numerical optimization and confirmation runs.

Model	Sorbent Dose (% *w*/*v*)		pH of Feed Solution		Response (Removal Efficiency, %)	
	*x*_1_ (Coded)	*SD* (Actual)	*x*_2_ (Coded)	pH (Actual)	${\hat{Y}}_{\mathrm{calculated}}$	$Y_{\mathrm{observed}}$
RSM	1.393	0.678	−0.365	6.4	88.05	85.93
ANN	1.409	0.682	0.073	7.1	85.53	86.18
SVM	1.372	0.674	0.007	7.0	84.95	86.28

## Data Availability

The data presented in this study are available on request from the corresponding author.

## Supplementary Materials

The following supporting information can be downloaded at: https://www.mdpi.com/article/10.3390/nano13040697/s1. Scheme S1: Greiss’ reaction employed for qualitative & quantitative analysis of nitrite ions (NO_2_^−^). (a) Classical Greiss’ reaction: under acidic conditions, NO_2_^−^ reacts with the amino moiety of sulfanilic acid to form diazonium cation, which links to α-naphthylamine in para-position to form the azo-dye identified by UV-Vis spectrophotometric method; (b) Modified Greiss’ reaction: under acidic conditions, NO_2_^−^ reacts with the amino moiety of sulfanilamide to form diazonium cation, which links to N-(1-naphthyl)ethylenediamine in para-position to form the azo-dye identified by UV-Vis spectrophotometric method [16]. Scheme S2: Artificial neuron structure and types of activation functions for multiple regression. Scheme S3: Representation of the principle of a support vector machine (SVM); Figure S1: Adsorption kinetics of (NO_2_^−^) ions onto nanoclay adsorbent (TmSA-MMT); T = 300 K, *SD* = 4 g/L, pH 7.0 ± 0.1, and [NO_2_^−^]_0_ = 5 mg/L; Figure S2: Adsorption equilibrium isotherm for the system NO_2_^−^/TmSA-MMT, at T = 300 K and initial pH of 7.0 ± 0.1; contact time *t* = 60 min; experimental observation: *q_e,max_* = 2.57 mg/g; data interpolation by Freundlich isotherm model (*K_F_* = 1.0391 and *n_F_* = 0.79627); Figure S3: Training performance of feed-forward ANN (2:3:1) model: Evolution of performance function (MSE) using LM-BP algorithm for training (goal = 5.00 × 10^−4^ and performance = 4.39 × 10^−4^); Figure S4: Molecular docking outcome: best pose of the docked complex showing the binding mode and interactions between TmSA (C_21_H_46_N^+^) (receptor) and NO_2_^−^ ion (ligand); computation results: binding affinity = −0.461 kcal/mol and dissociation constant *K_d_* = 459 mM; Figure S5: Probability plot of residuals (Normal distribution—95% Confidence Interval, CI); Figure S6: SEM images (cross-section view) of the produced flat-sheet porous membranes: (a,b) polymeric membrane (PVDF-HFP); (c,d) composite membrane (PVDF-HFP/HS); Figure S7: Histogram of pore size distribution in Layer-1 (skin-layer); Figure S8: Histogram of pore size distribution in Layer-2 (finger-like pores); Figure S9: Histogram of pore size distribution in Layer-3 (sponge-like pores); Figure S10: Histogram of pore size distribution (SEM top surface analysis); Figure S11: Water contact angle (WCA) measurements: (a) WCA = 80 ± 4° for polymeric membrane (PVDF-HFP); (b) WCA = 61 ± 3° for composite membrane (PVDF-HFP/HS); Figure S12: Estimation of permeate flux evolution for a longer time (*t* = 10,080 min, i.e., 168 h, or 7 d) by extrapolation using the computer-aided simulation based on the hyperbolic equation model; this simulation was performed to point out the meaning of the hyperbolic equation parameters; Table S1: Kinetic models and parameters for (NO_2_^−^) ions adsorption onto nanoclay adsorbent (TmSA-MMT), experimental conditions: T = 300 K, *SD* = 4 g/L, [NO_2_^−^]_0_ = 5 mg/L and pH 7.0; Table S2: Experimental design employed for Validation and Testing of ANN-model (Val and Test sets used for developing of ANN model); Table S3: Values of ANN-model parameters, i.e., weights (**IW**^(1,1)^, **LW^(^**^2,1)^) and biases ($\theta^{\left(1\right)}$**b**^)^,$\;\theta^{\left(2\right)}$*b*) for trained ANN (2:3:1), computed by LM-BP algorithm; Table S4: Values of least square SVM model parameters determined through LS-SVR; Table S5: Energy of intermolecular interactions between nitirite ion NO_2_^−^ as ligand and trimethyl stearyl ammonium TmSA (C_21_H_46_N^+^) as receptor; Table S6: ANOVA-Analysis of Variance (ANOVA) of the RSM-model; Table S7: ANOVA-Analysis of Variance (ANOVA) of the ANN-model; Table S8: ANOVA-Analysis of Variance (ANOVA) of the SVM-model (LS-SVM).
