# Supplementary material for: Application of Surface-Modified Nanoclay in a Hybrid Adsorption-Ultrafiltration Process for Enhanced Nitrite Ions Removal: Chemometric Approach vs. Machine Learning
Source: Nanomaterials (Basel). 2023 Feb 10;13(4):697. doi: 10.3390/nano13040697 (PMC9963183; doi:10.3390/nano13040697)
Supplement: Supplementary file 1 [file nanomaterials-13-00697-s001.zip › nanomaterials-2200985-supplementary.pdf]

nanomaterials

Supplementary Material

Application of Surface-Modified Nanoclay in a Hybrid Adsorption-Ultrafiltration Process for Enhanced Nitrite Ions Removal: Chemometric Approach vs. Machine Learning

Corneliu Cojocaru [1,*], Petronela Pascariu [2], Andra-Cristina Enache [1], Alexandra Bargan [1] and Petrisor Samoila [1]

1 Laboratory of Inorganic Polymers, "Petru Poni" Institute of Macromolecular Chemistry, 41A Grigore Ghica Voda Alley, 700487 Iasi, Romania
2 Laboratory of Physical Chemistry of Polymers, "Petru Poni" Institute of Macromolecular Chemistry, 41A Grigore Ghica Voda Alley, 700487 Iasi, Romania
* Correspondence: cojocaru.corneliu@icmpp.ro.

<u>Section S1.</u> Nitrite Ions Analysis and Adsorption Assays

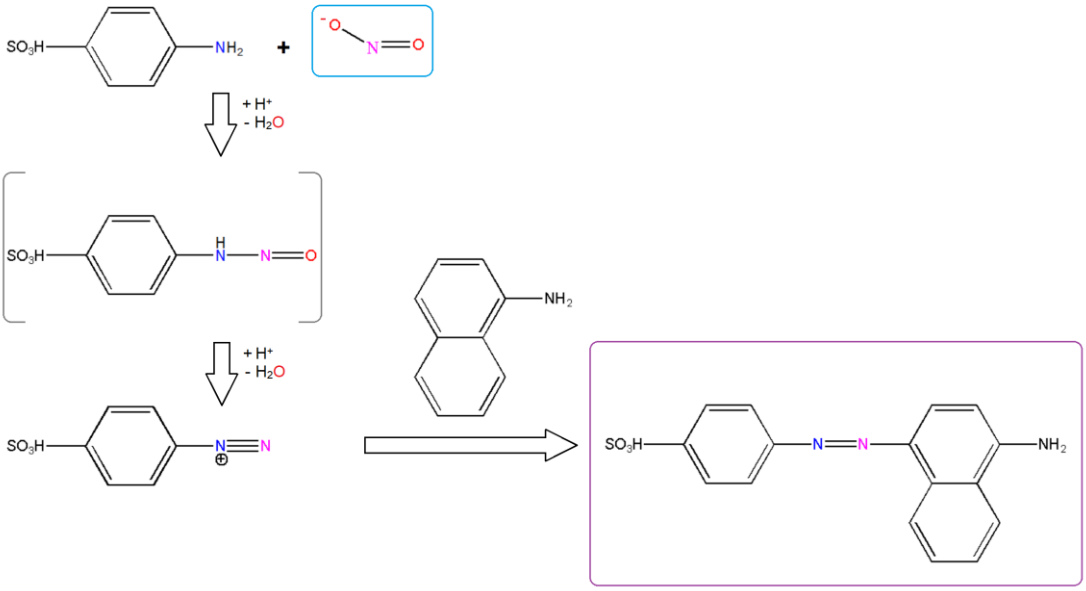

(a)

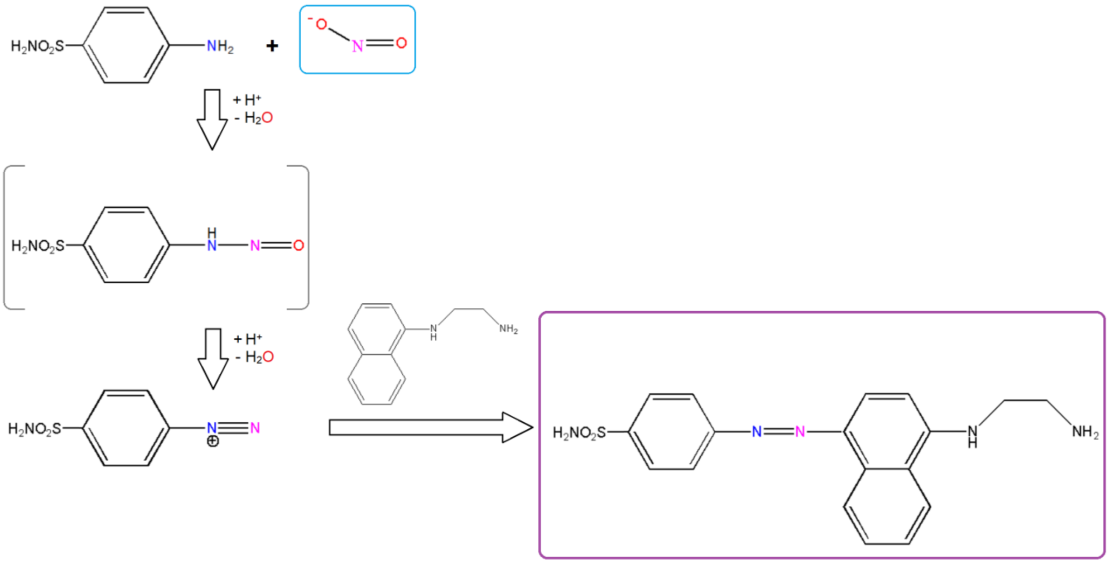

(b)

Scheme S1. Greiss' reaction employed for qualitative & quantitative analysis of nitrite ions (NO_2^-). (**a**) *Classical Greiss' reaction*: under acidic conditions NO_2^- reacts with the amino moiety of sulfanilic acid to form diazonium cation, which links to α-naphthylamine in para-position to form the azo-dye identified by UV-Vis spectrophotometric method; (**b**) *Modified Greiss' reaction*: under acidic con-ditions NO_2^- reacts with the amino moiety of sulfanilamide to form diazonium cation, which links to N-(1-naphthyl)ethylenediamine in *para*-position to form the azo-dye identified by UV-Vis spec-trophotometric method [16].

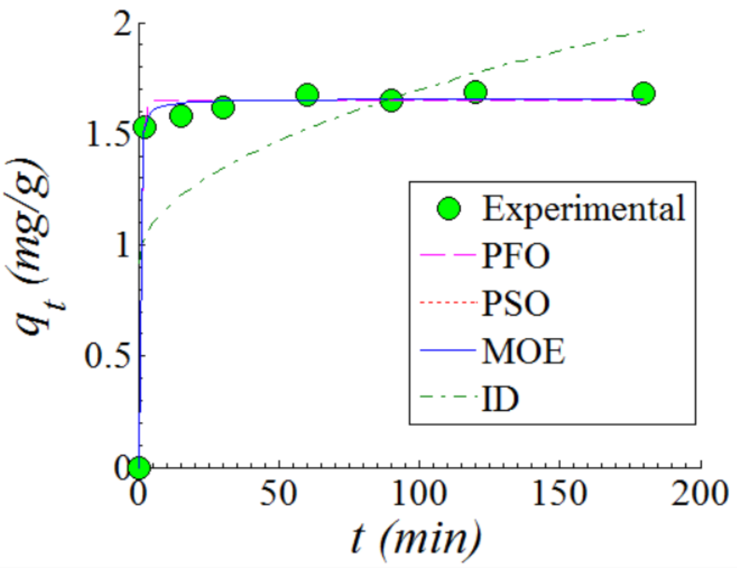

Figure S1. Adsorption kinetics of (NO_2^-) ions onto nanoclay adsorbent (TmSA-MMT); T = 300 K, *SD* = 4 g/L, pH 7.0 ± 0.1, and $[NO_2^-]_0$ = 5 mg/L

1. *note*: equilibrium attained for $t \geq 60$ min and $q_{e,obs}$ = 1.68 mg/g; kinetic data fitted to different kinetic models detailed in Table S1.

Table S1. Kinetic models and parameters for (NO_2^-) ions adsorption onto nanoclay adsorbent (TmSA-MMT), experimental conditions: T = 300 K, *SD* = 4 g/L, $[NO_2^-]_0$ = 5 mg/L and pH 7.0.

Model Abbreviation	Kinetic model (rate equation)	Kinetic model (non-linear equation)	Kinetic parameters
PFO [(a)]	$\frac{dq_t}{dt} = k_1(q_e - q_t)$	$q_t = q_e\left(1 - e^{-k_1 t}\right)$	q_e = 1.6487 (mg/g)<br>k_1 = 1.3157<br>χ^2 = 0.0052
PSO [(b)]	$\frac{dq_t}{dt} = k_2(q_e - q_t)^2$	$q_t = \frac{k_2 q_e^2 t}{1 + k_2 q_e t}$	q_e = 1.6572 (mg/g)<br>k_2 = 3.3637<br>χ^2 =0.0038
MOE [(c)]	$\frac{dq_t}{dt} = \sum_i K_i(q_e - q_t)^i$	$q_t = q_e \frac{1 - e^{(-K_1 t)}}{1 - \frac{K_2 q_e}{K_1 + K_2 q_e} e^{(-K_1 t)}}$	q_e = 1.6572 (mg/g)<br>K_1 = 4.4642 $\times 10^{-5}$<br>K_2 = 3.3636<br>χ^2 =0.0038
ID [(d)]	$\frac{dq_t}{dt} = \frac{k_d}{2\sqrt{t}}$	$q_t = k_d\sqrt{t} + J$	k_d = 0.078<br>J = 0.9171<br>χ^2 =0.4703

[(a)] PFO - pseudo-first order kinetics; [(b)] PSO - pseudo-second order kinetics; [(c)] MOE - mix 1,2-order kinetics; [(d)] ID - intra-particle diffusion kinetics.

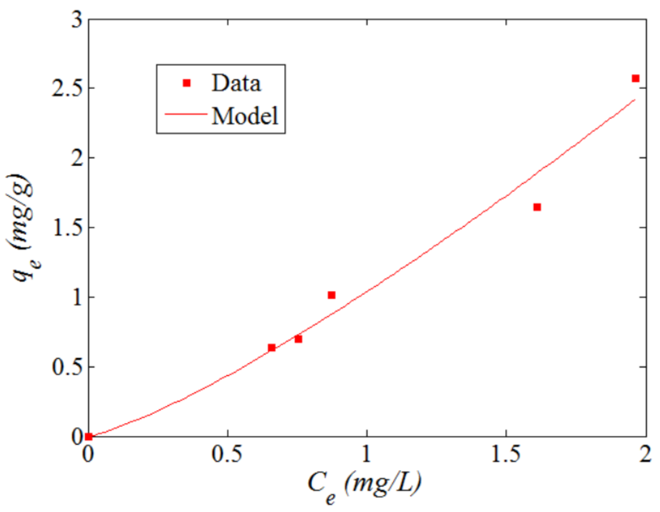

Figure S2. Adsorption equilibrium isotherm for the system NO_2^-/TmSA-MMT, at T = 300K and initial pH of 7.0 ± 0.1; contact time t = 60 min; experimental observation: $q_{e,max}$ = 2.57 mg/g; data interpolation by Freundlich isotherm model (K_F = 1.0391 and n_F = 0.79627).

Section S2. Response Surface Methodology RSM (Additional Details)

For modeling purpose, the real values of input variables (factors) are coded according to the following equation:

$$x_i = \frac{z_i - z_i^0}{\Delta z_i} \quad \text{(S1)}$$

where z denotes the real (actual) value of input variable; z^0 the center point of input variable (actual value); Δz the interval of variation; x the coded level of input variable (dimensionless value), and n is the number of input variables. Typically, the extent of each variable implies three different coded levels, from low (−1) to medium (0) and to high (+1). Additionally, depending on the type of the adopted experimental design, the axial points ($\pm\alpha$) can be also considered. The data-driven model provided by RSM represents a second-order polynomial equation with interaction terms that can be written as:

$$\hat{Y}_{RSM} = b_0 + \sum_{i=1}^{n} b_i x_i + \sum_{i=1}^{n} b_{ii} x_i^2 + \sum_{i<j}^{n} b_{ij} x_i x_j \quad \text{(S2)}$$

where: $\hat{Y}$ is the predicted response; x_i - coded levels of input variables; b_0, b_i, b_{ii}, b_{ij} are regression coefficients (intercept, main, quadratic and interaction effects), subscripts i and j are the integer indexes; and n is the number of design variables.

Section S3. Artificial Neural Network Modeling (Additional Details)

Neural network training can be more efficient if one performs certain *pre-processing* and *post-processing* steps on the network inputs and outputs [26].

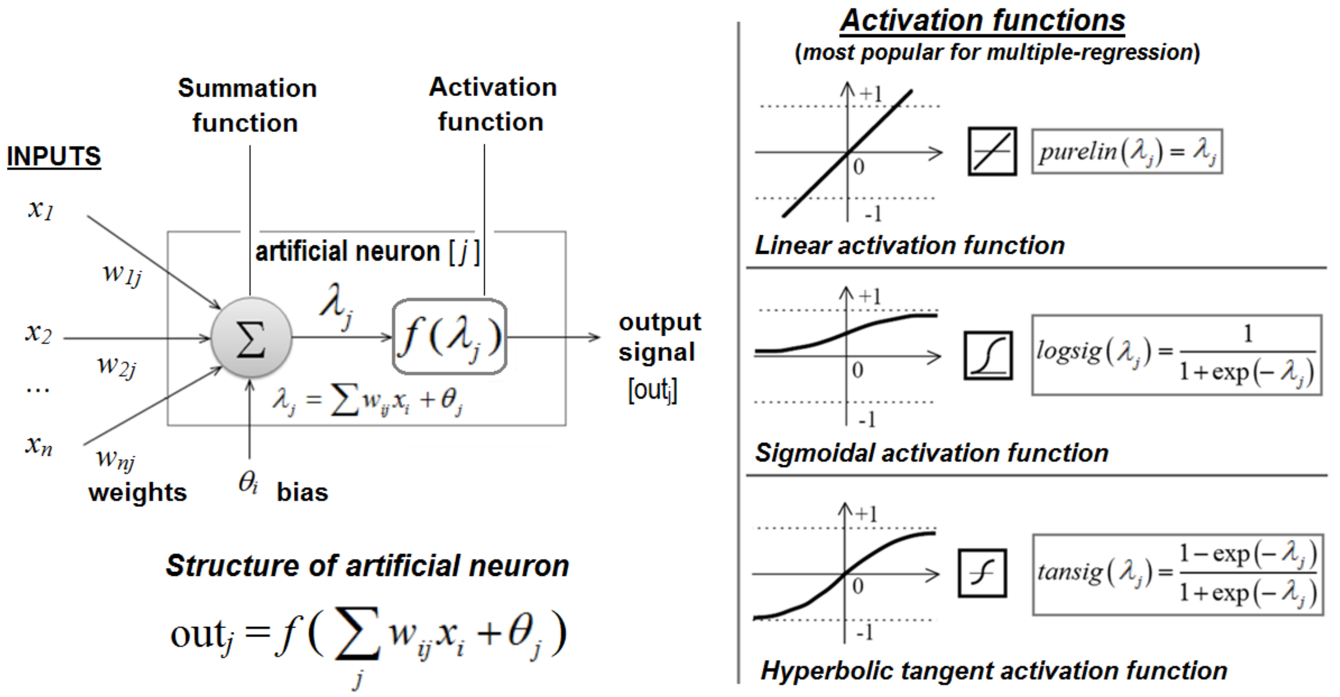

Scheme S2. Artificial neuron structure and types of activation functions for multiple regression.

Pre-Processing of Data (ANN Modeling)

The *pre-processing* step is carried out to avoid large over-fitting. In this section, the inputs **x** (for feeding the network) were normalized into the interval [-1.414 +1.414] using the same procedure as the one used for coding input variables in the RSM approach. In addition, the response (output variable or target) was normalized according to the following relationship:

$$Y_n = (1-2\Delta)\frac{(Y - Y_{\min})}{(Y_{\max} - Y_{\min})} + \Delta \quad \text{(S3)}$$

where Y_n denotes the normalized response (target), Y_{min} and Y_{max} designate the minimum and maximum levels of response in real values, and Y is the actual value of response. Note that, Δ is a small quantity set on 0.2 to ensure the normalized range of [0.2–0.8]. This reduced interval [0.2–0.8] offers to ANN a limited extrapolation capability for the extended region of [0–1].

Post-Processing of Data (ANN Modeling)

To switch back from normalized to actual values, one needs to apply the post-processing step. In this particular case, the following equation (S4) was applied to decode the response (output variable) from normalized to real values of outputs:

$$\hat{Y} = Y_{\min} + \frac{(\hat{Y}_n - \Delta)(Y_{\max} - Y_{\min})}{(1-2\Delta)} \quad \text{(S4)}$$

Also, to decode the input variable from its coded value to actual value the next relation was used:

$$z_i = z_i^0 + x_i \Delta z_i \quad \text{(S5)}$$

Table S2. Experimental design employed for *Validation* and *Testing* of ANN-model (*Val* and *Test* sets used for developing of ANN model).

Run	Sorbent dosage		Solution pH		Response	
	SD (%,w/v)	x_1	pH	x_2	*Y*, %	Y_n
Val-1	0.4	0	6.5	-0.333	81.07	0.717
Val-2	0.4	0	8.0	0.667	79.40	0.689
Val-3	0.6	1	6.5	-0.333	84.36	0.772
Test-1	0.4	0	6.0	-0.667	80.00	0.699
Test-2	0.4	0	7.5	0.333	80.41	0.706

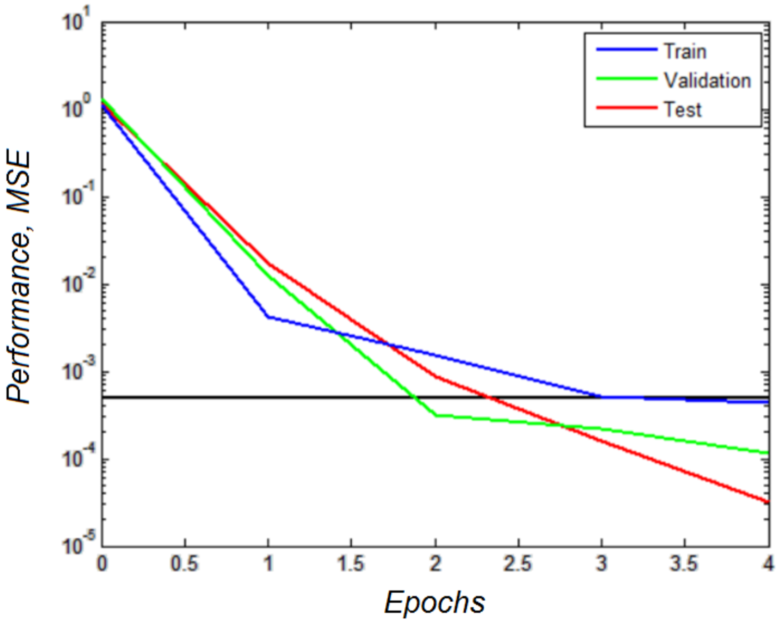

Figure S3. Training performance of feed-forward ANN (2:3:1) model: Evolution of performance function (*MSE*) using LM-BP algorithm for training (*goal* = 5.00 × 10^{-4} and *performance* = 4.39 × 10^{-4}).

Table S3. Values of ANN-model parameters, *i.e. weights* (**IW**$^{(1,1)}$, **LW**$^{(2,1)}$) and *biases* ($\boldsymbol{\theta}^{(1)}$**b**), $\theta^{(2)}b$) for trained ANN (2:3:1), computed by LM-BP algorithm.

**Input weight matrix, IW$^{(1,1)}$ {Destination: Hidden layer Source: Inputs}**	$\mathbf{IW}^{(1,1)} = \begin{bmatrix} -0.0427 & -3.7778 \\ -3.9731 & 1.1413 \\ -1.4243 & -1.5313 \end{bmatrix}$
**Bias vector, $\boldsymbol{\theta}^{(1)}$** {Destination: Hidden layer}	$\boldsymbol{\theta}^{(1)} = \begin{bmatrix} 5.4105 & -1.2229 & -3.3764 \end{bmatrix}^T$
**Layer weight vector, LW$^{(2,1)}$** {Destination: Output layer Source: Hidden layer}	$\mathbf{LW}^{(2,1)} = \begin{bmatrix} 0.2139 & -0.2524 & -0.8432 \end{bmatrix}$
Bias scalar, , $\theta^{(2)}$ {Destination: Output layer}	$\theta^{(2)} = 0.5829$

2. *Note*: Reported *weights* and *biases* refer to normalized values of inputs and output.

Section S4. Support Vector Machine (SVM) (Additional Details):

The support vector machine (SVM) and its algorithm for regression (*i.e.*, support vector regression SVR) rely on Vapnik's ε-*intensive loss function* applied in statistical learning theory. The basic principle of SVM is depicted in the scheme below (adapted after [29]):

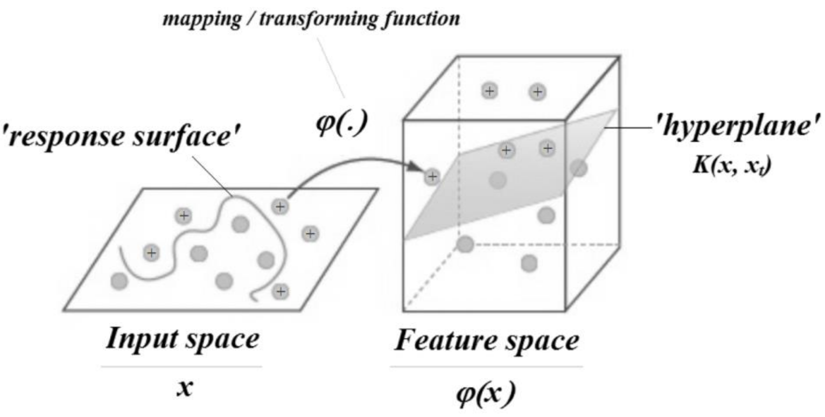

Scheme S3. Representation of the principle of a support vector machine (SVM).

Generic relation in SVM (and LS-SVM) used for response function estimation can be written as:

$$\hat{Y}_{\mathrm{SVM}} = f(\mathbf{x}) = \sum_{\tau=1}^{n_\tau} \alpha_\tau K(\mathbf{x},\ \mathbf{x}_\tau) + \beta = \sum_{\tau=1}^{n_\tau} \alpha_\tau \left(\varphi(\mathbf{x})^{\mathrm{T}} \varphi(\mathbf{x}_\tau)\right) + \beta = \sum_{\tau=1}^{n_\tau} \alpha_\tau \exp\left(\frac{-\left(\|\mathbf{x}-\mathbf{x}_\tau\|^2\right)}{\sigma^2}\right) + \beta \quad \text{(S6)}$$

where,

$\mathbf{x} = \begin{bmatrix} x_1 & x_2 \end{bmatrix}^{\mathrm{T}}$ - vector of input variables used to estimate the response $\hat{Y}_{\mathrm{SVM}}$

$\mathbf{x}_\tau = \begin{bmatrix} x_{1,\tau} & x_{2,\tau} \end{bmatrix}^{\mathrm{T}}$; $\forall \tau = \overline{1, n_\tau}$ - vector of input variables from the training set;

τ - integer index, and n_τ – number of data points in the training set; $K(\mathbf{x},\mathbf{x})$ – *Kernel* function;

φ - irregular function for mapping input data to high-dimensional space (feature space).

Table S4. Values of least square SVM model parameters determined through LS-SVR.

No.	*Parameters of LS-SVM model with RBF kernel :* **LS-SVM**$^{\mathrm{RBF}}_{\gamma=10,\ \sigma^2=0.2}$
1	$\boldsymbol{\alpha} = [-1.7590\ \ 0.7212\ \ -0.3544\ \ 0.8277\ \ -0.9335\ \ 0.9898\ \ 0.0473\ \ -0.2080\ \ 0.6688]^T$
2	$\beta = 0.0044$
3	*tuning parameters in LS-SVM:* σ^2 = 0.2 (*squared bandwith*); γ = 10 (*regularization parameter);*

Section S5. Molecular Docking Simulation

In addition, we investigated by computer-aided simulation the molecular docking between NO_2^- oxoanion (considered as *ligand*) and *trimethyl stearyl ammonium* TmSA ($C_{21}H_{46}N^+$) as *receptor*. The structures of ligand and receptor were constructed in YASARA-Structure program [39]. The molecular docking simulations were performed by using the AutoDock-VINA algorithm [40] included in YASARA-Structure software (v.20.8.23) [39]. The results of molecular docking are given in **Figure S4** and **Table S5**. Hence, molecular

docking simulation suggested that the retention of the nitrite ion by TmSA is mainly based on the electrostatic interactions (*i.e.*, Coloumb forces, see **Table S5**).

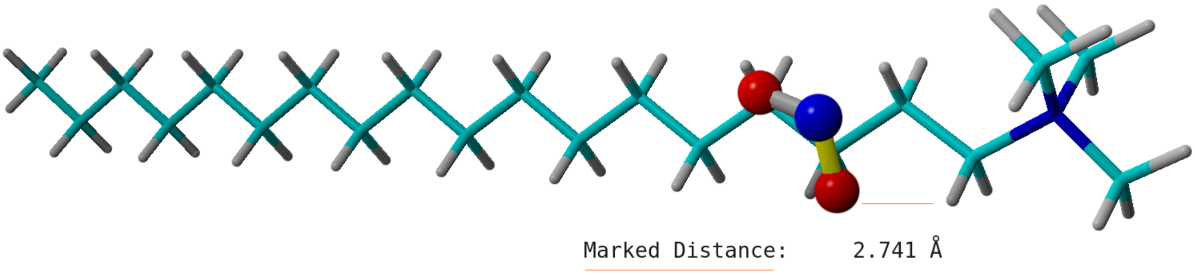

Figure S4. Molecular docking outcome: best pose of the docked complex showing the binding mode and interactions between TmSA ($C_{21}H_{46}N^+$) (receptor) and NO_2^- ion (ligand); computation results: *binding affinity* = −0.461 kcal/mol and *dissociation constant* K_d = 459 mM.

Table S5. Energy of intermolecular interactions between nitirite ion NO_2^- as ligand and trimethyl stearyl ammonium TmSA ($C_{21}H_{46}N^+$) as receptor.

Docking system (ligand/receptor)	*Total intermolecular:* $\Delta E = \Delta E_{VdW} + \Delta E_{CL}$, (kcal/mol)	*Van-der-Waals:* ΔE_{VdW}, (kcal/mol)	*Coulomb:* ΔE_{CL}, (kcal/mol)
NO_2^-/TmSA	−52.81 (100%)	−2.84 (5.38%)	−49.97 (94.62%)

<u>Section S6</u>. Analysis of variances (ANOVA) and inspection of residuals

Table S6. ANOVA-Analysis of Variance (ANOVA) of the RSM-model.

Source	*DF* [(a)]	*SS* [(b)]	*MS* [(c)]	*F-value* [(d)]	*P-value* [(e)]	R^2 [(f)]	$R_{adj}{}^2$ [(g)]
Model	5[(*)]	1205.83	241.17	15.34	0.0047	0.938	0.877
Residual	5	78.61	15.72				
Total	10						

[(a)] degree of freedom; [(b)] sum of squares; [(c)] mean square; [(d)] ratio between mean squares;

[(e)] probability of randomness; [(f)] coefficient of determination; [(g)] adjusted coefficient of determination.

[(*)] For calculation of DF associated to Model source, the number of model parameters was considered equal to n_L = 6 ($DF_1 = n_L-1$) being associated to number of RSM-model parameters: {b_0, b_1, b_2, b_{11}, b_{22}, b_{12}}.

Table S7. ANOVA-Analysis of Variance (ANOVA) of the ANN-model.

Source	*DF*	*SS*	*MS*	*F-value*	*P-value*	R^2	$R_{adj}{}^2$
Model	3[(**)]	1399.07	466.36	297.36	<0.0001	0.989	0.986
Residual	10	15.68	1.57				
Total	13	1414.75					

[(**)] For calculation of DF associated to Model source, the number of model parameters was considered equal to n_L=4 ($DF_1 = n_L-1$) being associated to ANN-model weights and biases: $\mathbf{IW}^{(1,1)}$, $\mathbf{LW}^{(2,1)}$, $\mathbf{b}^{(1)}$, and $b^{(2)}$.

Table S8. ANOVA-Analysis of Variance (ANOVA) of the SVM-model (LS-SVM).

Source	*DF*	*SS*	*MS*	*F-value*	*P-value*	R^2	$R_{adj}{}^2$
Model	2[(***)]	1274.06	637.03	491.10	<0.0001	0.992	0.990
Residual	8	10.38	1.30				
Total	10	1284.44					

(***) For calculation of DF associated to Model source, the number of model parameters was considered equal to n_L=3 (DF_1 = n_L−1) being associated to SVM-model parameters, *i.e.* α, *β*, and *γ*.

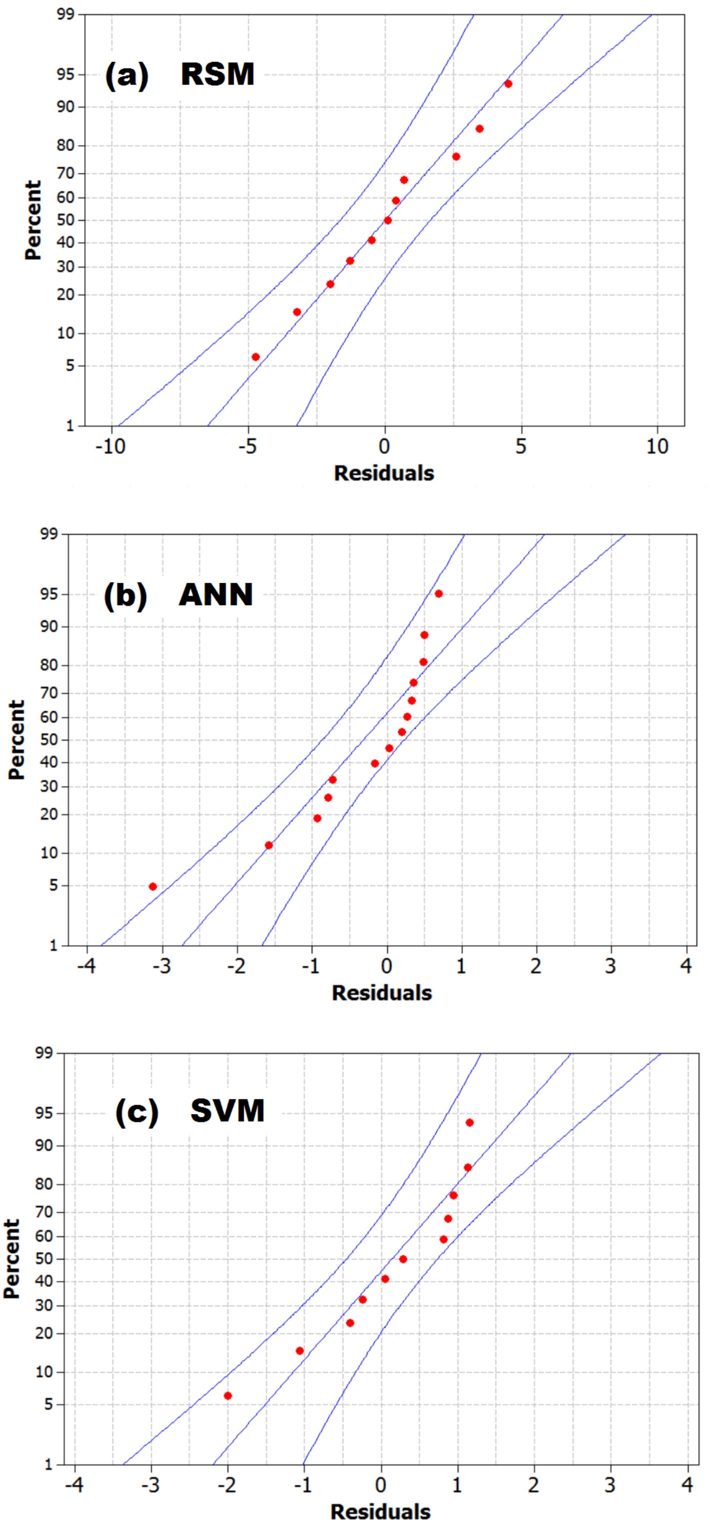

Figure S5. Probability plot of residuals (Normal distribuiton – 95% Confidence Interval, CI).

<u>Section S7</u>. Composite Membrane (PVDF-HFP / Halloysite)

Preparation of the Composite Membrane (PVDF-HFP / Halloysite)

A new composite membrane, made of poly(vinylidene fluoride-co-hexafluoropropylene) (PVDF-HFP) and Halloysite nanoclay (HS), was prepared by the phase inversion method using DMAc (ρ = 0.937 g/mL) as solvent and PEG (400 Da, ρ = 1.128 g/mL) as a porogen agent. The main steps of the preparation are detailed in the next. First, PVDF-HFP pellets (1.75 g) and PEG (0.62 mL) were added to 80% of DMAc volume (6.4 mL). Afterwards, the resulting mixture was stirred at 65 ^{o}C and 200 rpm in an orbital-shaker incubator (BIOSAN ES-20/60) until PVDF-HFP was completely dissolved. This polymeric solution (DMAc/ PVDF-HFP /PEG) was denoted hereafter as *solution-I*. Second, the Halloysite particles (0.055 g) were dispersed in the remaining 20% of DMAc volume (1.6 mL) under ultrasonication (45 min). The obtained dispersed solution was denoted as *solution-II*. Third, the nanoparticle dispersion (*solution-II*) was then added to the polymeric *solution-I*, and the resulted casting solution (PVDF-HFP/PEG/DMAc/HS) was ultrasonicated for 30 minutes. The final weight content of components in the casting solution was equal to 17.5% wt. (PVDF-HFP), 7.0% wt. (PEG), 0.5% wt. (HS) and 75.0% wt. (DMAc). Ultimately, the casting solution was poured on a glass plate and stretched as a thin layer of 300 μm thickness, using a film applicator (ZUA 2000, ZEHNTNER). After about 10 seconds of air-exposure of the casting film, the glass plate was immersed into a coagulation bath containing distilled water. This immersion (in non-solvent) promoted the phase-inversion process, done at room temperature. The formed membrane was peeled off, rinsed and maintained in distilled water for about 24 h. Then, the flat-sheet membrane was dried for 48 h and stored between two filter papers, before testing.

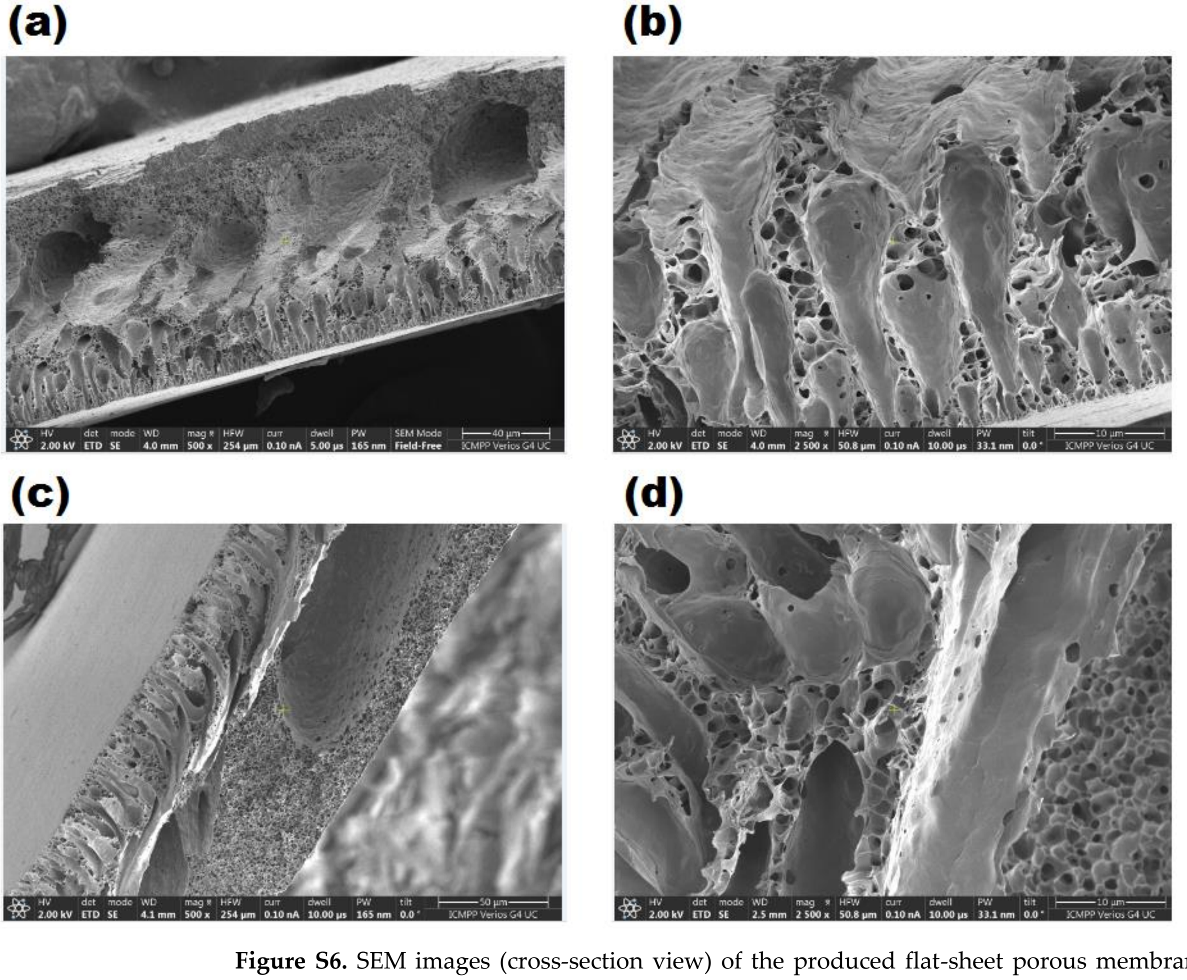

Figure S6. SEM images (cross-section view) of the produced flat-sheet porous membranes: (**a**,**b**) polymeric membrane (PVDF-HFP); (**c**,**d**) composite membrane (PVDF-HFP/HS).

Pore Size Distribution: Histogram Analysis & Lognormal Distribution

Pore sizes were measured from SEM images in order to determine their distribution by histogram analysis. In statistics, when building a histogram, the *skewness* represents the degree of asymmetry observed in a probability distribution. Hence, the distributions may exhibit right (positive) skewness or left (negative) skewness to varying degrees. The ***normal distribution*** (bell curve) exhibits zero skewness, whereas the ***lognormal distribution*** is a distribution skewed to the right. According to the *lognormal distribution*, the probability density function (PDF) starts at zero, increases to its mode, and decreases thereafter. The most important *lognormal statistical* functions are listed in the following:

Lognormal Probability Distribution Function (Lognormal-PDF, f_{LN}):

$$f_{LN} = \frac{1}{h\,\sigma_\mu\sqrt{2\pi}}\exp\left(-\frac{(\ln(h)-\mu)^2}{2\sigma_\mu^2}\right) \quad \text{(S7)}$$

Lognormal Cumulative Distribution function (Lognormal-CDF, F_{LN}):

$$F_{LN} = \frac{1}{2}\left[1+\operatorname{erf}\left(\frac{\ln(h)-\mu}{\sigma_\mu\sqrt{2}}\right)\right] \quad \text{(S8)}$$

***Skewness* (*Sk*) for lognormal distribution:**

$$Sk = \left(\exp(\sigma_\mu^2)+2\right)\sqrt{\exp(\sigma_\mu^2)-1} \quad \text{(S9)}$$

where, h – pore size (µm), μ - location parameter of lognormal distribution; and σ_μ - scale parameter of lognormal distribution.

Pore sizes measured from SEM images were subjected to distribution analysis by building the corresponding histograms. According to the developed histograms (**Figures S7–S10**), the distributions are skewed to the right. Therefore, the *lognormal distribution* functions (PDF & CDF) were developed for the considered sets of pore sizes.

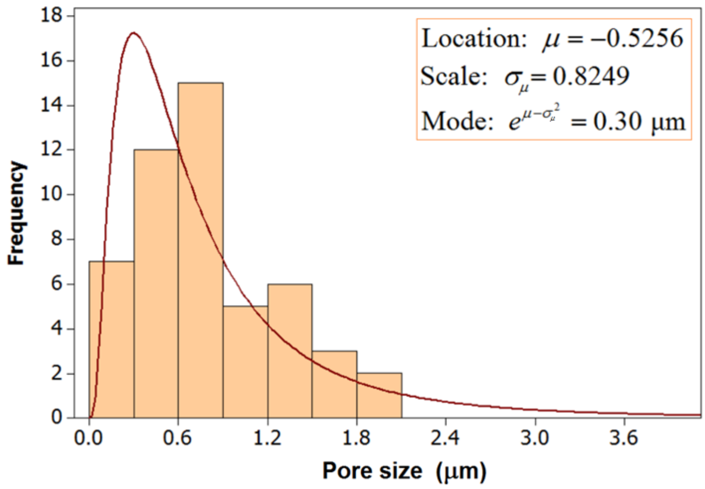

Figure S7. Histogram of pore size distribution in Layer-1 (*skin-layer*).

Descriptive Statistics: Size = 50 (number of data points); Min = 0.05 µm, Max = 1.98 µm, Median = 0.68 µm; Mean = 0.76 µm; StdDev = 0.48 µm; *Sk* = 3.924.

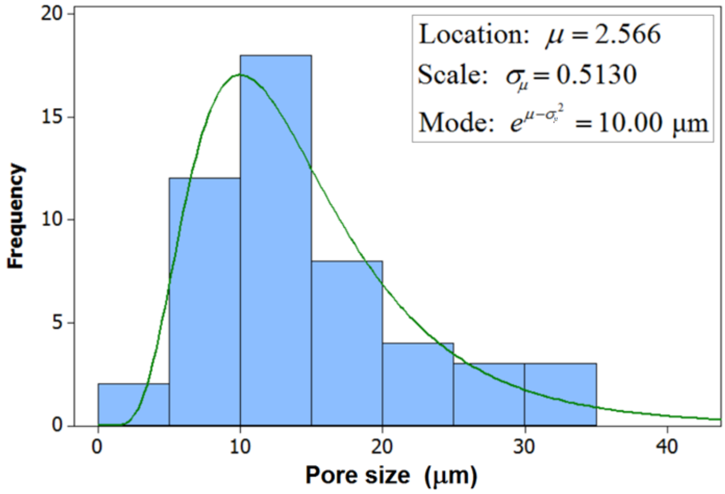

Figure S8. Histogram of pore size distribution in Layer-2 (*finger-like* pores).

Descriptive Statistics: Size = 50 (number of data points); Min = 3.98 μm, Max = 34.50 μm,
Median = 13.24 μm; Mean = 14.74 μm; StdDev = 7.47 μm; *Sk* = 1.811.

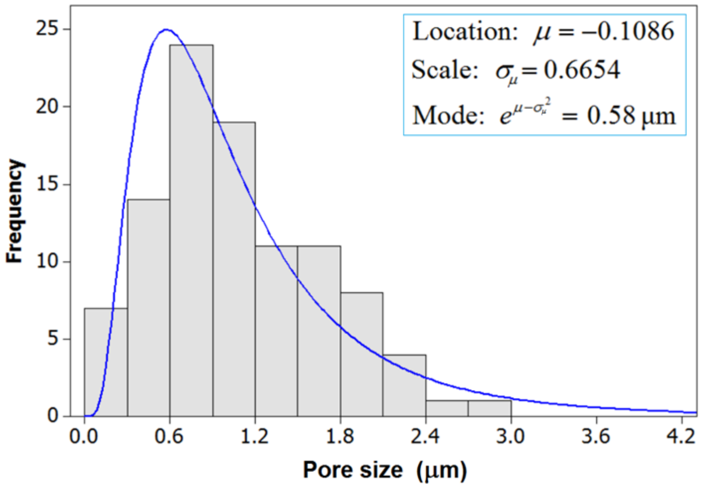

Figure S9. Histogram of pore size distribution in Layer-3 (*sponge-like* pores).

Descriptive Statistics: Size = 100; Min = 0.10 μm, Max = 2.76 μm, Median = 0.97 μm; Mean = 1.08 μm; StdDev = 0.60 μm; *Sk* = 2.655.

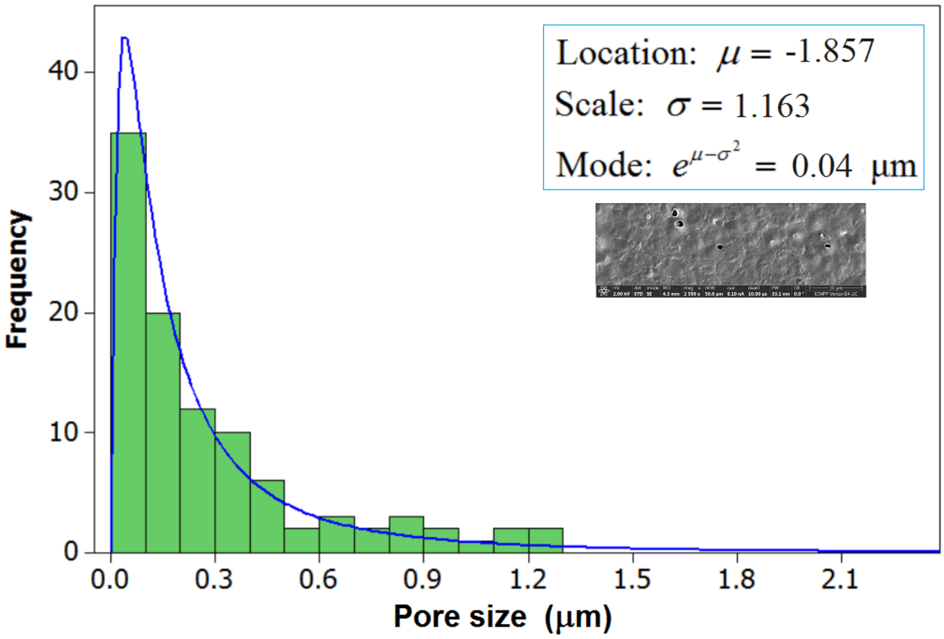

Figure S10. Histogram of pore size distribution (SEM top surface analysis).

Descriptive Statistics: Size = 100; Min = 0.01 μm, Max = 1.25 μm, Median = 0.15 μm; Mean = 0.30 μm; StdDev = 0.77 μm; *Sk* = 9.935.

Porosity of the Composite Membrane

The porosity (ε, %) of the porous membrane represents the volume of pores (voids) divided by the total volume of the membrane. In our case, the porosity of the produced membrane was determined gravimetrically by immersion of this material in the liquid phase of *isopropyl alcohol*, and measuring the weight of liquid contained in the pores [48]:

$$\varepsilon = \frac{V_L}{V_L + V_M} \times 100 = \frac{(w_+ - w_0)/\rho_L}{(w_+ - w_0)/\rho_L + w_0/\rho_M} \times 100 \quad \text{(S10)}$$

where, V_L (cm³) – volume of liquid trapped in pores (“equivalent” to the volume of pores); V_M (cm³) – volume of the solid material (membrane - excluding pores); w_+ (g) - weight of the wet porous material (containing the trapped liquid in pores); w_0 (g) - weight of the dry porous material; ρ_L (g/cm³) – density of the liquid (*isopropyl alcohol*); and ρ_M (g/cm³) - density of material/polymer. Hence, for the produced composite membrane (PVDF-HFP[97%]/HS[3%]) the measured porosity was equal to **ε =75.32 ± 2.65%.**

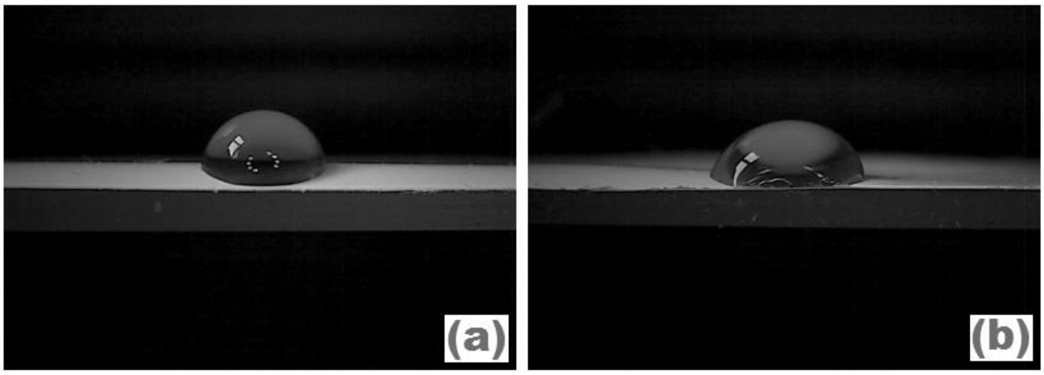

Figure S11. Water contact angle (WCA) measurements: (**a**) WCA = 80 ± 4° for polymeric membrane (PVDF-HFP); (**b**) WCA = 61 ± 3° for composite membrane (PVDF-HFP/HS).

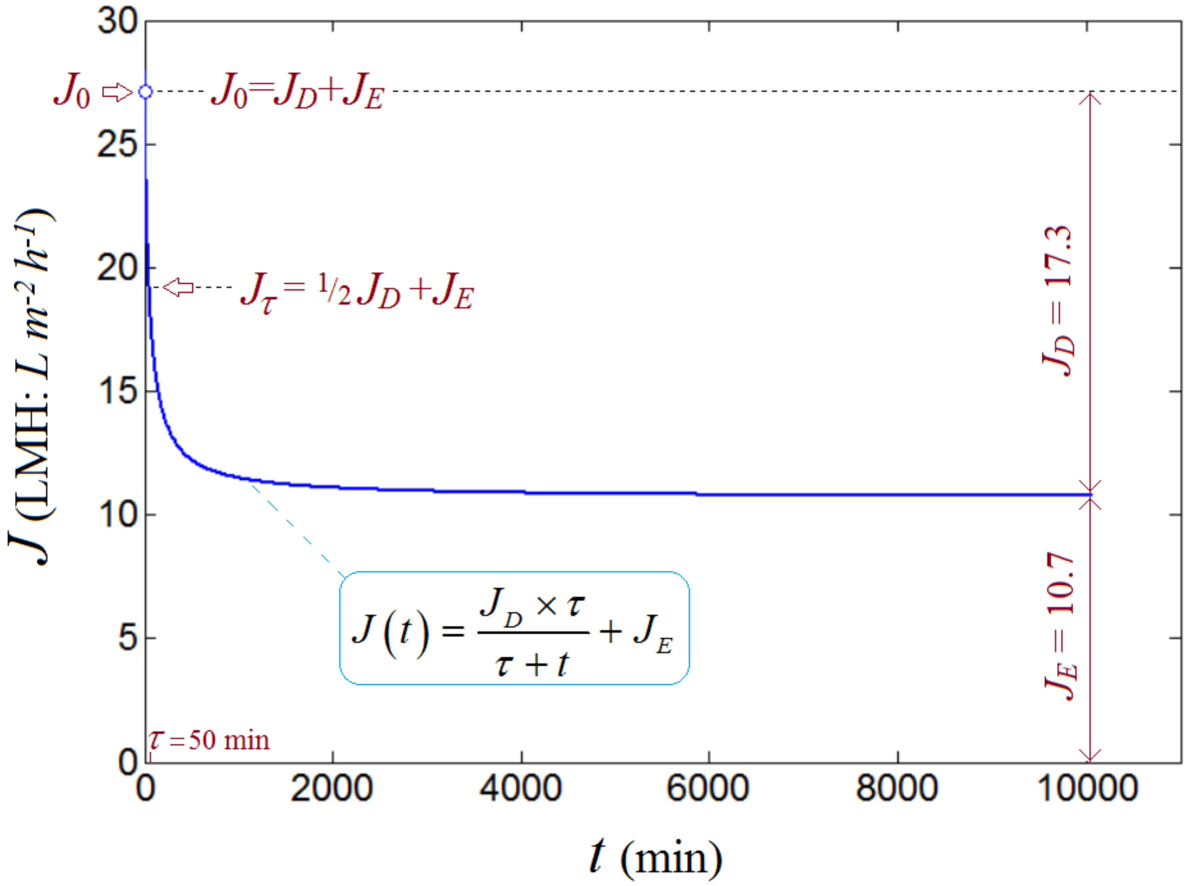

Figure S12. Estimation of permeate flux evolution for a longer time (t = 10080 min, *i.e.*, 168 h, or 7 d) by extrapolation using the computer-aided simulation based on the hyperbolic equation model; this simulation was performed to point out the meaning of the hyperbolic equation parameters.

Section S8. FTIR Spectra Analysis: Assignments of IR Peaks

For interpreting infrared spectra, the abbreviations ν, δ , and ω were used to indicate the *stretching*, *bending*, and *wagging* mode vibrations, respectively. Main assignments of infrared (IR) wave-numbers for the investigated materials are summarized in the following.

Halloysite nanotubes (HS) – the HS sample was pressed into KBr pellets for IR analysis:
ν(O-H): 3694, 3622, and 3551 cm^{-1} (inner-surface hydroxyl, inner hydroxyl, and adsorbed water molecules, respectively); δ(O-H): 1651 cm^{-1} (O-H deformation vibration of the water adsorbed); ν(Si-O): 1118 cm^{-1}; ν(Si-O-Si): 1029 cm^{-1}; δ(Al-OH): 908 cm^{-1}; δ(Si-O-Al): 750, 686, and 538 cm^{-1} (bending deformation).

PVDF-HFP (in ATR mode): ν(C-H): 3023–2853 cm^{-1}; ω(CH_2, CH_3) + ν(C-F): 1401 cm^{-1} (CH_2 and CH_3 wagging deformation overlapped with C-F stretching vibration); ν(C-F): 1180 and 1069 cm^{-1} (C-F stretching vibration); δ(C-C): 879 and 838 cm^{-1} (C-C skeleton vibration associated to the amorphous phase of polymer); δ(C-C): 765 and 976 cm^{-1} (α-crystal phase of polymer); ω(CF_2): 681 and 612 cm^{-1} (CF_2 wagging vibration mode).

PVDF-HFP/HS composite membrane (in ATR mode): ν(O-H): 3694 and 3625 cm^{-1} (from HS); ν(C-H): 3023–2853 cm^{-1}; ωCH_2, CH_3) + ν(C-F): 1401 cm^{-1} (CH_2 and CH_3 wagging deformation overlapped with C-F stretching vibration); ν(C-F): 1180 and 1069 cm^{-1} (C-F stretching vibration); δ(C-C): 976 cm^{-1} (α-crystal phase of polymer); δ(C-C): 879 and 838 cm^{-1} (C-C skeleton vibration associated to the amorphous phase of polymer); δ(C-C) + δ(Si-O-Al): 765 cm^{-1}; δ(Si-O-Al) + ω(CF_2): 686 cm^{-1}; ω(CF_2): 612 cm^{-1} (CF_2 wagging vibration mode).
